# Mafic-ultramafic igneous rocks as a source of reactive phosphorus for the origin of life

**DOI:** 10.1038/s41467-026-75933-6

**Published:** 2026-07-23

**Authors:** Abu Saeed Baidya, Craig R. Walton, Joanna Kalita, Kristoffer Szilas, Marco Viccaro, Paul S. Savage, Stuart Allison, Euan G. Nisbet, Maria Schönbächler, Sami Mikhail, Eva E. Stüeken

**Affiliations:** 1 School of Earth and Environmental Sciences, University of St. Andrews, St Andrews, UK; 2 Department of Earth and Planetary Sciences, ETH Zurich, Zurich, Switzerland; 3 Trinity College, University of Cambridge, Cambridge, UK; 4 Natural History Museum of Denmark, University of Copenhagen, Copenhagen, Denmark; 5 Department of Biological, Geological and Environmental Sciences, University of Catania, Catania, Italy; 6 National Institute of Geophysics and Volcanology, Section of Catania – Etnean Observatory, Catania, Italy; 7 Department of Earth Sciences, Royal Holloway, University of London, Egham, UK

**Keywords:** Astrobiology, Geochemistry, Mineralogy, Geochemistry

## Abstract

Phosphite and pyrophosphate—reduced and polymerized forms of phosphorus—may have played a central role in prebiotic chemistry owing to their higher reactivity and solubility, yet their geological sources remain uncertain. Here we show that mafic–ultramafic rocks on the early Earth could have provided a sustained source of reactive phosphorus through seafloor weathering. Rock analyses from 15 localities reveal phosphite comprising up to 7%, 24%, 17%, and 0.6% of total phosphorus in olivine separates, peridotite, komatiite, and basalt, respectively, while pyrophosphate accounts for up to 5% and 0.4% in komatiite and basalt. Box-model calculations indicate phosphite concentrations reaching up to 1 µM in the deep ocean and up to 67 µM in lakes under low-UV conditions. We suggest that reactive phosphorus released from mafic–ultramafic rocks could have fuelled prebiotic phosphorylation and supplied phosphate to emerging life on Earth, a process that may also enhance the habitability of other planetary bodies.

## Abstract (web summary)

Mafic–ultramafic rocks on early Earth may have supplied reactive phosphorus via seafloor weathering. Models suggest concentrations may have been sufficient to support prebiotic chemistry and life on Earth and enhance habitability on other planetary bodies.

## Introduction

Phosphorus (P) is indispensable for life as a structural component of nucleic acids, cell membranes, and adenosine triphosphate (ATP). Among the major bioessential elements, however, P is the least abundant in surface environments^1^ and has no stable gaseous phase, limiting its availability for prebiotic chemistry on terrestrial planets. Under most habitable surface conditions, P occurs as orthophosphate (P(V) or P_i_), an oxidized, sparingly soluble, and relatively inert species^2^. While this stability is key for the persistence of biological molecules, it hinders prebiotic phosphorylation reactions^3^. High concentrations of P_i_ may have been required for life’s origins, yet such environments were likely rare^4^.

Alternatively, prebiotic chemistry may have drawn on more soluble and reactive P species, particularly phosphite (P(III)) and pyrophosphate (PP(V) or PP_i_)^5–7^. For example, PP_i_ can act as a phosphorylating agent^8^, a precursor to ATP-based metabolism, and a potential energy source for early life^9^. On the other hand, P(III) is ca. 1000 times more soluble than P_i_ in natural fluids, and more efficient at producing phosphorylated organics^6,7,10^. Furthermore, geochemical analyses of Paleoarchean carbonates and Neoarchean–Paleoproterozoic banded iron formations indicate that P(III) comprised potentially 5–88% of dissolved inorganic P in the Archaean ocean^11,12^.

Two main abiotic mechanisms have been proposed for forming these reactive and soluble P species. The first involves the aqueous dissolution of iron–nickel phosphides (e.g., schreibersite, (Fe,Ni)₃P), delivered by meteorites^13^ or formed during contact-metamorphism^14,15^ and lightning strikes^16^. Schreibersite corrosion releases P(III), PP_i_, and other reactive and soluble P compounds^17–19^. The second mechanism involves thermal processing of P_i_ precursors in magmatic or metamorphic settings. Volcanic processes above ~1200 °C can produce P_4_O_10_, which hydrates to PP_i_ and other higher-order polyphosphates; such species have been detected in volcanic fumaroles in Japan^20^. Dry-heating of alkali, ammonium, or divalent metal P_i_ salts can yield polyphosphates including PP_i_, with higher yields in the presence of Fe–Cr–Ni-bearing minerals^7,21,22^. Metamorphic heating of phosphate precursors with Fe^2+^, H_2_, or organic matter can also reduce P_i_ to P(III), as shown by dry-heating of amorphous Fe-phosphate, where Fe^2+^ oxidation accompanies P_i_ reduction^7,14,21^. Phosphite detected in Eoarchean carbonates and iron formations of amphibolite–granulite grade may reflect such metamorphic reduction^7,21^. Collectively, these studies indicate that low water activity, high temperature, and reducing conditions promote P(III) generation.

Although the mechanisms for P(III) and PP_i_ formation described above seem feasible, they are spatially and temporally limited, episodic, or restricted in scale, raising concerns about their ability to sustain widespread and continuous prebiotic P chemistry. In this study, we reveal a previously understudied geochemical source of reactive P species: magmatic mafic and ultramafic rocks and their associated fluid–rock interactions. These lithologies, which were volumetrically extensive on the early Earth and remain common on other planetary bodies, are characterized by high formation temperatures (usually >1000 ^o^C), and variable but often low water activity and high Fe^2+^ concentrations that should favour the formation and stabilization of reduced and polymerized P species.

To evaluate this hypothesis, we analyzed several mafic to ultramafic rocks representing the top and bottom of the prebiotic lithosphere, from 15 globally distributed locations (Supplementary Table 1, Supplementary Fig. S1). We chose basalt and komatiite because they were the dominant surface rocks on the prebiotic and early Earth. Peridotite, particularly harzburgite and dunite, were included to probe the mantle reservoir, as they represent refractory residues formed after partial mantle melting that has led to the formation of basalt and komatiite melts. Ultramafic rocks in particular are undersaturated with respect to apatite, meaning that olivine, which can contain several wt% P^23^, would be the main reservoir of P in these rocks so long as the *f*O_2_ was above the iron-iron wüstite buffer^24^ below which native Fe and thus iron phosphides such as FeP and Fe_3_P are stabilised. Hence, we also investigated olivine separates from lherzolite peridotite, a pallasite meteorite, and basalt. Phosphorus was extracted from rock powders with an EDTA–NaOH solution (solid-to-solution ratio 1:10, shaken for 14–15 hours at room temperature), and concentrations of P(III), P_i_, and PP_i_ were quantified by ion chromatography coupled with inductively coupled plasma mass spectrometry (IC–ICP–MS; see Methods)^11,25^. Separate powder aliquots were analyzed for total P and major and trace element concentrations by bulk digestion and ICP-MS. Data from basalts in the Moodies Group (Barberton, South Africa) were taken from a previous study^15^. The compiled data were used to estimate the potential reserves of reactive P species—particularly P(III) and PP_i_—in the early Earth’s oceanic crust. We further use these data to constrain a simple box model of the prebiotic ocean and of a volcanic lake where rock weathering would have liberated P(III) into solution.

Our findings suggest that: (1) Earth’s crust in the Hadean to Archaean may have contained reactive P species up to 0.70% of total P (with 0.34 % P(III) and 0.34% PP_i_); (2) early Earth mafic and ultramafic lithologies could have provided sustained sources of reactive P for prebiotic phosphorylation and early metabolic pathways; and (3) similar lithologies present on other planetary bodies such as Mars and Enceladus may harbour primary reactive P reservoirs suitable for prebiotic chemistry.

## Results and Discussion

### Phosphorus speciation in mafic and ultramafic rocks

We find that the total P content is lowest in peridotite (10-40 ppm with an average of 17 ppm). The olivine separate from lherzolite contains a similarly low amount of P (20 ppm). Komatiite is P-enriched compared to peridotite (70–120 ppm, average 93 ppm), and basalt shows even higher enrichments (590-4110 ppm, average 2097 ppm) (Supplementary Dataset 1). The olivine separate from basalt is P-depleted (40 ppm) compared to bulk average basalts, suggesting that most P in these basalts is not olivine-hosted and instead perhaps present as apatite or contained in other silicate minerals or glass. Although collected from genetically unrelated settings, total P in the studied samples shows a good correlation with K_2_O (r^2^ = 0.77, Supplementary Fig. S3A). Overall, these trend reflect the incompatible behaviour of P during mantle melting and magmatic differentiation, which leads to progressive enrichment in differentiating melts until apatite saturation is reached^26,27^. Komatiite forms due to a higher degree of partial melting of the mantle compared to basalt^28^, which explains enhanced P concentrations in the lower degree melts (basalt) compared with higher degree melts (komatiite).

**Fig. 1:**
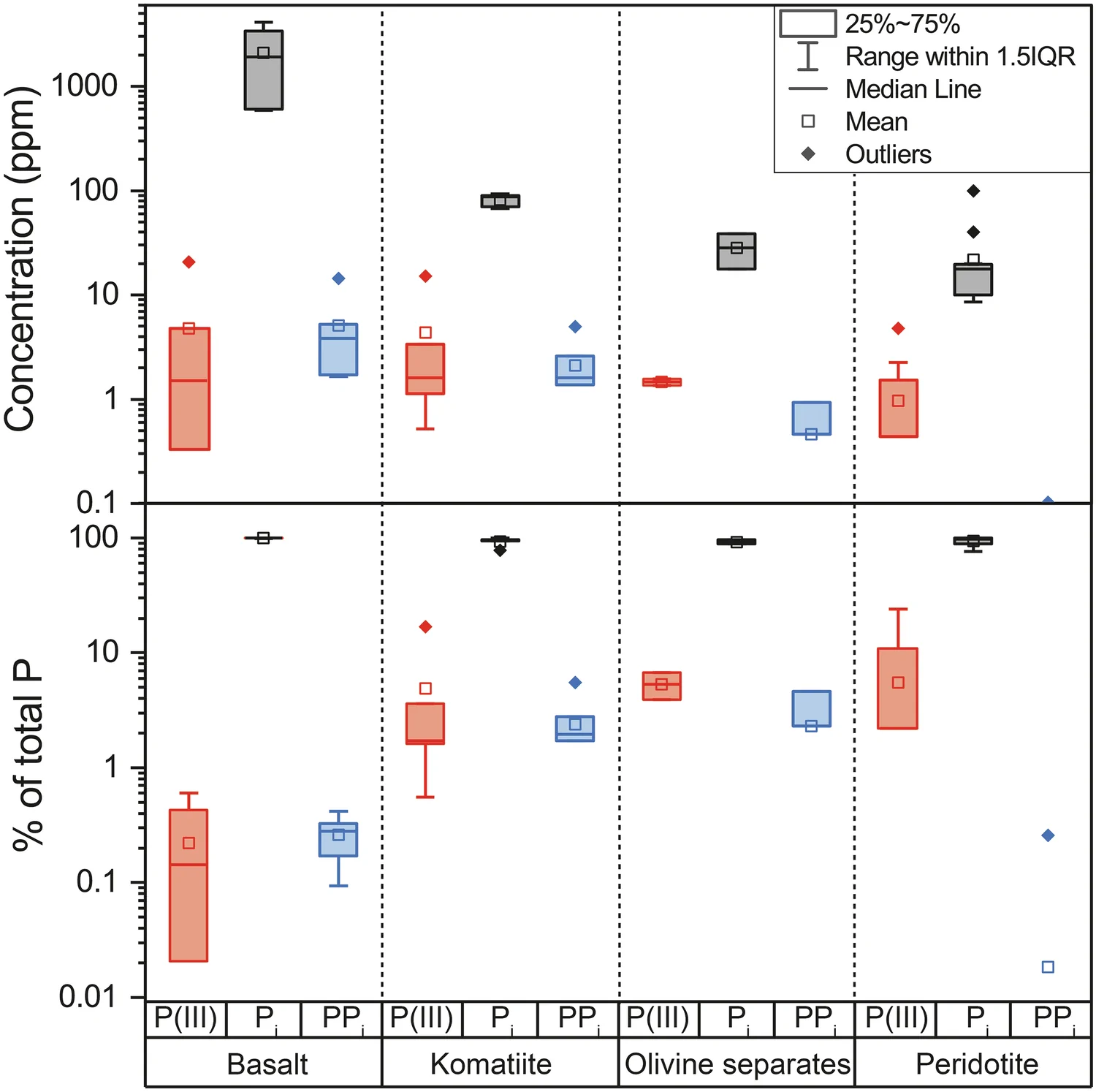
Abundance and speciation of phosphorus in mafic and ultramafic rocks. Bottom panel: Box plots showing the relative abundance of phosphorus species—phosphite (P(III)), phosphate (P(V)), and pyrophosphate (PP_i_)—expressed as a percentage of total extracted P in EDTA–NaOH solutions. The whiskers extend to 1.5 × the interquartile range (IQR) from the lower and upper quartiles; points beyond this range are plotted as outliers. Komatiite, olivine, and peridotite samples exhibit higher proportions of P(III) compared to basalt, while PP_i_ is enriched in komatiites but largely absent in olivine and peridotite. Top panel: Estimated concentrations of each P species in bulk rock (ppm), calculated using total P content, extraction yield, and speciation data from the EDTA–NaOH extracts. Phosphate concentrations are highest in basalt and decrease progressively in komatiite, olivine, and peridotite. In contrast, P(III) concentrations are broadly similar across all lithologies.

Our P speciation analyses reveal that mafic and ultramafic rocks are important repositories of reduced and polymerized P species, particularly P(III) and PP_i_. The IC-ICP-MS measurements of EDTA–NaOH extracts indicate that komatiite, olivine separates, and peridotite samples contain markedly higher proportions of P(III) relative to the total P content compared to basalt. The average percentages of P(III) in basalt, komatiite, olivine separates, and peridotite are 0.22%, 4.89%, 5.33%, and 5.52%, respectively, relative to total P (Table 1, Fig. 1). Several factors may influence this pattern, including formation temperature, the compatibility of P(III) during melting and magmatic differentiation, and mantle-related controls such as redox state, silicate host phases, and the possible presence of phosphide.

**Table 1:** Concentrations of phosphorus species in the studied mafic and ultramafic rocks

Rock types	Average proportions (%)						Average Concentration (ppm)*					
	P(III)	SD P(III)	P(V)	SD P(V)	PP_i_	SD PP_i_	P(III)	SD P(III)	P(V)	SD P(V)	PP_i_	SD PP_i_
Basalt (*n* = 6)	0.22	0.24	99.52	0.33	0.26	0.11	4.78	7.95	2086.79	1447.80	5.09	4.73
Komatiite (*n* = 5)	4.89	6.82	92.71	8.74	2.40	2.03	4.37	6.17	81.52	11.94	2.11	1.86
Olivine separates (*n* = 2)	5.33	1.99	92.35	5.27	2.31	3.27	1.46	0.16	28.08	14.64	0.46	0.65
Peridotite (*n* = 15)	5.52	7.05	94.46	7.03	0.02	0.07	0.97	1.34	21.69	23.04	0.01	0.03
	﻿**Median proportions (%)**						**Median Concentration (ppm)**					
Basalt (*n* = 6)	0.14		99.63		0.28		1.51		1912.93		3.83	
Komatiite (*n* = 5)	1.71		96.43		1.96		1.60		87.35		1.60	
Olivine separates (*n* = 2)	3.06		96.94		0.00		1.46		28.08		0.46	
Peridotite (*n* = 15)	2.20		97.80		0.00		0.44		17.75		0.00	

*Concentrations are estimated using ratios of P-species in the EDTA-NaOH extract, extraction yield, and total P contents. *SD* standard deviation of the average concentrations.

Previous experiments and thermodynamic equilibrium modelling have suggested that, in the presence of Fe(II), elevated temperatures (200–700 °C) favour the reduction of P_i_ to P(III), thereby increasing the equilibrium P(III)/ΣP ratio^7,21^. Experiments under magmatic conditions have so far not been conducted; however, if this trend holds, then komatiitic magmas, which form at substantially higher temperatures than basaltic magmas (ca. >1600 °C versus <1200 °C)^29,30^, may also express a higher P(III)/ΣP equilibrium ratio. Formation temperature could therefore play a role in establishing higher P(III)/ ΣP ratios in komatiites compared to basalts.

Alternatively, or in addition, it is possible that the trends are driven by compatibility of P(III). For basalts and komatiites, P(III)/ΣP shows modest but systematic relationships with the differentiation-sensitive trace elements La and Ni (Supplementary Fig. S6). Because La behaves incompatibly and is enriched in lower-degree basaltic melts, whereas Ni is associated with more primitive, olivine-rich ultramafic systems, the negative correlation between P(III)/ΣP and La and the positive correlation with Ni are consistent with relatively greater retention of a reduced-P component in olivine-rich residues and in melts derived from them. P(III) may thus be relatively more compatible with mafic minerals than P_i_. Under this interpretation, basaltic melts generated by lower degrees of mantle melting contain a much larger oxidized-P reservoir relative to reduced P, whereas komatiitic melts generated by higher degrees of mantle melting may inherit proportionally more reduced P from an olivine-rich mantle source. This interpretation is also consistent with the relatively high P(III) abundances observed in dunite and olivine separates. In basalt, whole-rock P is high, but the basalt-hosted olivine separate is P-poor relative to the bulk rock, indicating that most basaltic P is hosted outside olivine, as stated above. By contrast, peridotites and olivine separates are low-P, olivine-rich residues, so even a modest amount of reduced P retained in olivine would constitute a much larger fraction of the total P inventory. Accordingly, P(III)/ΣP is high in peridotites, komatiites, and olivine separates, but low in basalt: in ultramafic, olivine-rich rocks the total P reservoir is small, so any preserved reduced-P component becomes proportionally important, whereas in basalt the much larger oxidized P reservoir dilutes the P(III) fraction.

Further, mantle redox may control the P(III)/ΣP ratio in the ultramafic dataset. The upper-mantle oxygen fugacity is heterogeneous over several log units, from about FMQ − 3 to FMQ + 1, with some cratonic Archaean lithosphere extending even lower^31,32^. Our peridotite and olivine separates come from multiple mantle sources and tectonic settings, so some may be derived from reduced mantle domains that may contribute to high P(III)/ΣP. However, we did not see any trend; for example, Kilbourne Hole peridotite with a much higher *f*O_2_ (FMQ + 0.4^33^) compared to the Jiaohe peridotite (*f*O_2_ = FMQ − 1.9 to FMQ−1.3^34^) and Balmuchia peridotite (*f*O_2_ = FMQ^35^), shows a much higher P(III)/ΣP ratio (14%) compared to the latter two peridotites (P(III)/ΣP ≈ 0). Furthermore, source redox is unlikely to explain the komatiite data from the Belingwe Greenstone belt, because Fe³⁺/ΣFe ratios in melt inclusions of these komatiites indicate a source with an oxidation state broadly similar to present-day MORB-source mantle^36^. Therefore, redox potential probably does not play an important role for the studied samples but should be explored further in the future.

A further possibility, particularly relevant to komatiitic magmas, is that deeper and higher-degree melting entrained small phosphide-bearing reduced-P domains from the mantle source. Phosphides have been reported from mantle-hosted podiform chromitites, showing that highly reduced P domains can occur in mantle settings^37^. If such phases were present in the source of the Belingwe komatiites, their entrainment into ascending melts and partial oxidation during eruption and cooling in the presence of H₂O could have contributed to the phosphite detected here. Overall, the elevated P(III)/ΣP in peridotite and olivine separates is best explained by preservation of reduced P in olivine-rich, low-P ultramafic residues and possibly by elevated temperatures, whereas the higher P(III)/ΣP in komatiite relative to basalt likely reflects a combination of this silicate-host effect with deeper melting and, potentially, contribution from phosphide-bearing reduced domains.

Regarding PP_i_, komatiite and basalt samples exhibit moderate abundances (2.40% and 0.26%, respectively, relative to total P), whereas this species is below the detection limit in olivine and peridotite (Table 1, Fig. 1). We speculate that PP_i_ behaves like an incompatible species, such that it becomes enriched during mantle melting. This inference is supported by strong correlations between PP_i_ and molybdenum (Mo) as well as potassium (K), both of which behave incompatibly during partial melting and show an enrichment in basalt compared to ultramafic rocks and minerals and correlate with total P content (Supplementary Fig. S5). These elements are selected because K is a widely used major-element indicator of incompatible behaviour during igneous processes, whereas Mo provides a trace-element comparison with similarly incompatible behaviour. This positive relationship suggests that as residual melts become enriched in P, this increases the probability of phosphate–phosphate interactions and thus PP_i_ formation. These observations are consistent with experimental results showing that polyphosphates can form in heated mixtures of basalt and apatite at temperatures above the basalt melting point (~1200 °C), where the enhanced mobility of P_i_ in the melt was proposed to promote polymerization reactions^20^.

To estimate the concentrations of different P species in bulk samples, we normalized the speciation data to the extraction yield, determined by comparison to the total P contents. This approach is justified through the lack of any relation between P extraction yields and P(III)/ΣP ratio, which indicates that there was no preferential extraction of P(III) and P_i_ (Supplementary Fig. S7). It also follows the conventions of previous studies^11^. These calculations show that P(III) concentrations remain relatively constant across the studied lithologies (average of 4.78 ppm, 4.37 ppm, 1.46 ppm, and 0.97 ppm for basalt, komatiite, olivine separates, and peridotite, respectively), while P_i_ concentrations follow the same pattern as total P (Fig. 1). The basalt samples, although they display lower P(III)/ ΣP ratios than komatiites and ultramafic rocks, can thus still yield comparable absolute P(III) concentrations because their total P contents are substantially higher. Absolute concentrations of PP_i_ in basalt and komatiite are also similar (5 ppm and 2 ppm, respectively; Fig. 1). We note that our IC–ICP–MS measurements quantify dissolved P(III) in the EDTA–NaOH extract, but they do not distinguish whether the reduced P was originally present in the rock as a protonated P(III) species (e.g. HPO_3_^2–^, H_2_PO_3_^–^, H_3_PO_3_) or a less protonated P(III) ion (PO_3_^3-^). As the EDTA-NaOH solution is highly alkaline (pH is around 14), and the stability of protonated P(III) species is highly dependent on pH, all extracted P(III) species are converted to their most deprotonated form. D-label solvent and NMR analysis (^1^H and ^31^P) may resolve the degree of primary protonation in the future, particularly if the concentration in the extraction solution is higher than >200 ppb. We discard the possibility of mechanochemical reduction of polyphosphates into phosphite^38,39^ as we did not use any reducing agent during the EDTA-NaOH extraction; the extraction was done in the presence of oxygen and water, which prohibit phosphate reduction^21^, and apart from PP_i_ no other polyphosphate was detected.

The observed distribution of reduced and polymerized P species reflects primary magmatic processes rather than secondary alteration. P(III) has been reported from serpentinized ultramafic rock, and it has been suggested that the serpentinization process may facilitate P_i_ reduction to P(III)^40^. However, the studied komatiite samples show only minor alteration^41^. Similarly, the olivine separates and peridotite samples do not show any visible alteration, and the positive correlation between CaO and Al₂O₃ is consistent with preservation of primary igneous/mineralogical compositional variations, rather than secondary alteration (Supplementary Figs. S1 and S2). Some basalt samples, particularly from the Moodies Group, show minor alteration in the form of secondary sericite formation; however, altered samples were not analysed in this study^15^. Furthermore, K_2_O is positively correlated with U in the basalt samples, indicating that the incompatible and fluid-mobile element geochemistry is controlled by magmatic factors and not controlled by secondary processes (Supplementary Fig. S1D). Importantly, fluid-induced alteration would imply the addition of water; however, experimental studies have suggested that elevated water activity prohibits the formation of reduced and polymerized P species from phosphate percursors^7,21,22^. Therefore, P(III) and PP_i_ are unlikely to be the product of secondary alteration and serpentinization. Furthermore, a recent study noted magmatic PP_i_ in two natural olivine samples^42^ while experimental studies suggest that magmatic glass may host polyphosphates^43^. We therefore conclude that the observed P(III) and PP_i_ are magmatic in origin. We further suggest that, as serpentinites formed by hydration and alteration of ultramafic precursor rocks, it is plausible that at least part of the P(III) that has previously been reported from serpentinites^40^ was inherited from reduced phosphorus already present in the precursor lithologies and subsequently redistributed or released during serpentinization.

Basalt and komatiite may have represented the principal magmatic sources of reactive P species on the early Earth. Although the relative abundances of P(III) and PP_i_ are lower in basalts compared to ultramafic rocks, their substantially higher total P contents result in comparable absolute concentrations of reactive P species (Fig. 1). Moreover, both basalt and komatiite are highly susceptible to chemical weathering and commonly contain volcanic glass, which alters more rapidly than mineral phases^24^. These properties make basaltic and komatiitic lithologies not only efficient hosts for reactive P but also more accessible contributors of magmatic reactive P species via fluid-rock interaction, underscoring their potential significance in prebiotic geochemical cycles.

### Reactive phosphorus reservoirs in Earth’s oceanic crust

**Fig. 2:**
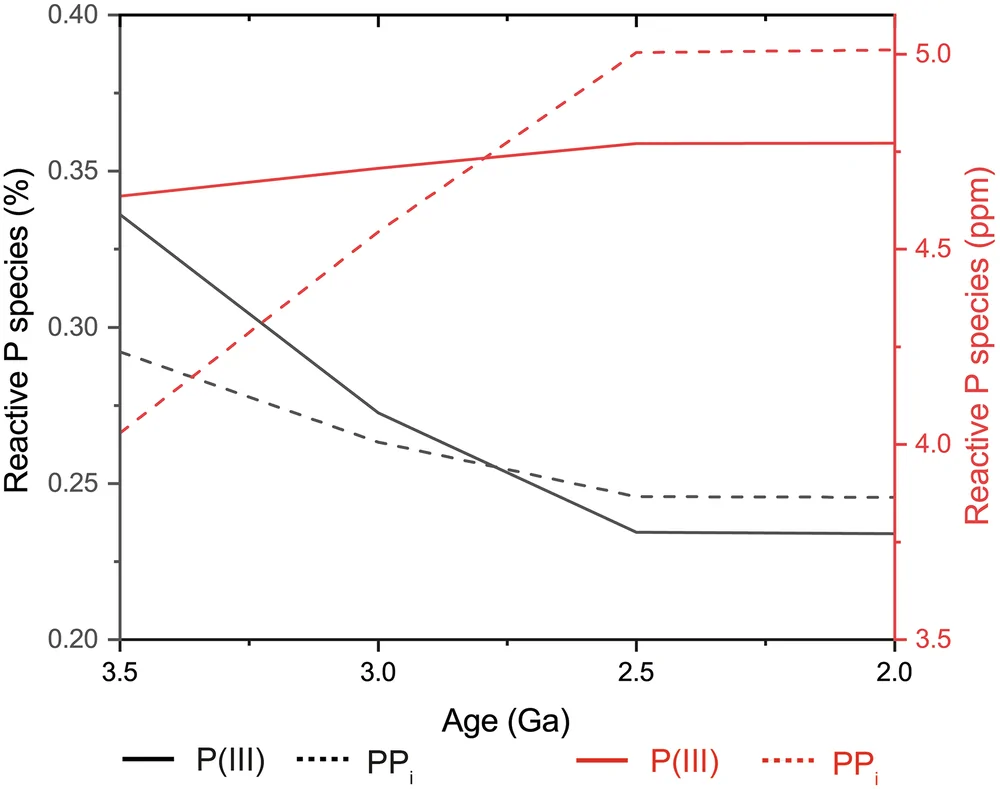
Oceanic crustal reservoir of reactive phosphorus species. Temporal evolution (3.5–2.0 Ga) of phosphite (P(III)) and pyrophosphate (PP_i_) in the oceanic crust, shown as both relative proportions (%) and absolute concentrations (ppm). Black lines (left y-axis) indicate the relative abundances of P(III) and PP_i_ in %, while red lines (right y-axis) denote their estimated concentrations in ppm. Estimates are based on the evolving proportion of basalt and komatiite in the crust^44^ and the measured concentrations of P(III) and PP_i_ in these lithologies (see Method section for details). The declining trend in reactive P proportions is attributed to the progressive decrease in komatiite content over time. Absolute concentrations of both species in the oceanic crust remain between 4 and 5 ppm across this time interval.

The P speciation data in the studied samples allow us to evaluate the oceanic crustal reservoir of reactive P species through time. We considered the evolving composition of the oceanic crust between 3.5 Ga and 2.0 Ga, integrating the declining abundance of komatiite and increasing dominance of basalt^44^ with their respective reactive P profiles (see Method section for details; Fig. 2a). Herein, we do not consider the continental crust reservoir of reactive P species, which requires additional studies. Our emphasis on oceanic crust, which is most relevant for a prebiotic scenario with limited extent of continents on the early Earth, and it is also relevant to other terrestrial planets such as Mars where felsic continental crust does not occur. Our calculations for the 3.5–2.0 Ga timeframe show similar values of relative abundance and absolute concentration of P(III) and PP_i_ in the oceanic crust such that relative percentages of P(III) and PP_i_ vary between 0.33–0.22% and 0.28–0.22%, respectively, whereas the absolute concentrations of these two species vary between 4–5 ppm during this interval (Fig. 2a). Importantly, these results suggest that oceanic crust and seamount volcanoes could have been a long-term and stable source of reactive and soluble P species via seafloor weathering.

### Phosphite availability in early ocean and lake environments

**Fig. 3:**
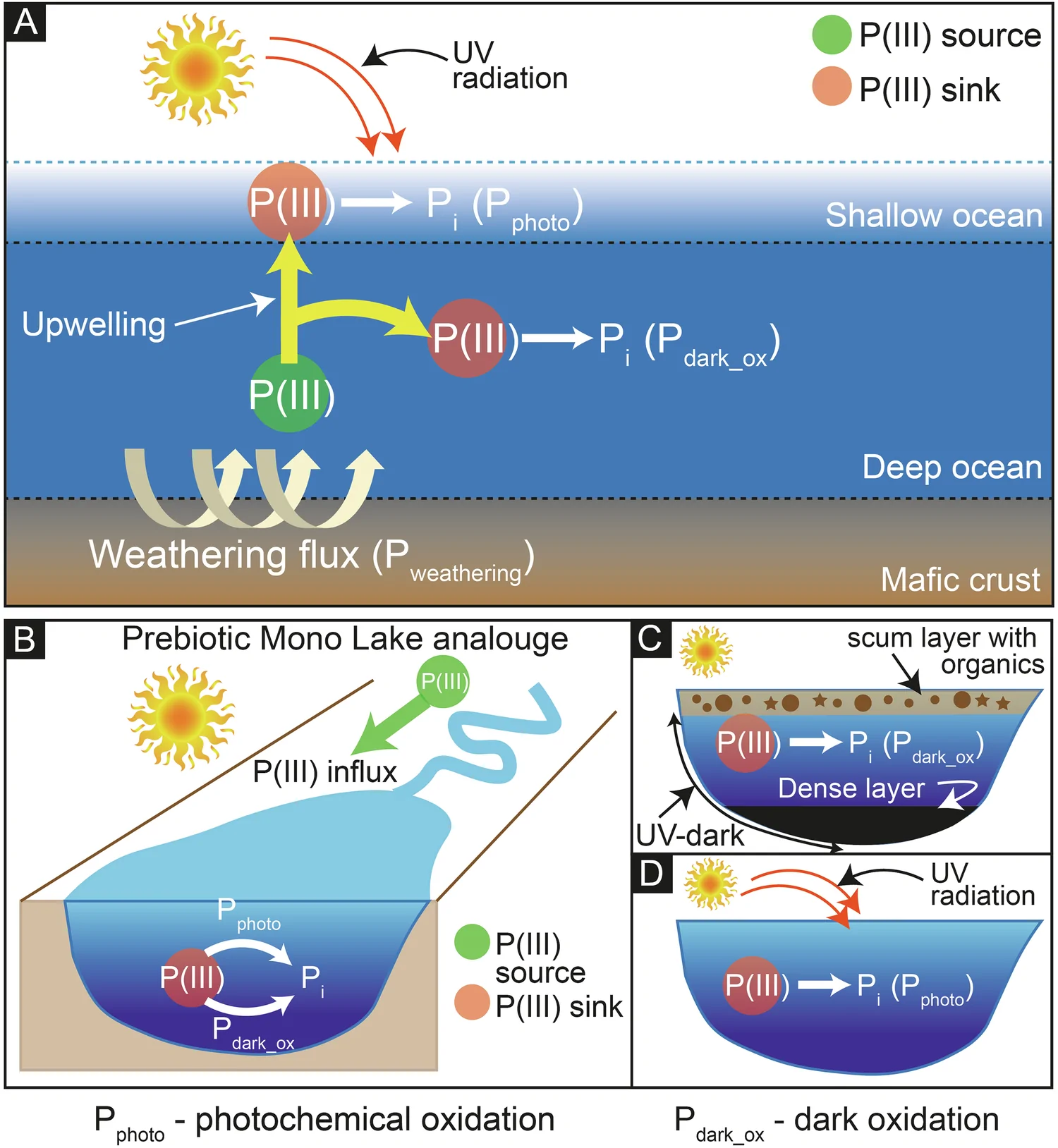
Conceptual models for phosphite (P(III)) cycling in early Earth ocean and lake environments. **A** Schematic representation of the box model components used to estimate P(III) concentrations in the deep ocean. The primary source is ocean floor weathering, while major sinks include photochemical oxidation in the sunlit surface ocean (driven by UV radiation) and dark oxidation in deeper waters. **B**–**D** Box models for estimating P(III) concentrations in a prebiotic Mono Lake analogue (model adapted from Walton et al.^4^). Riverine input is the dominant source, while photochemical and dark oxidation serve as sinks. (**C**) A stratified lake scenario in which UV radiation is attenuated, either by the formation of an organic-rich surface scum^24^ or by density stratification^4^, resulting in reduced P(III) oxidation and higher concentrations. **D** An unstratified lake with full UV exposure, leading to enhanced photochemical oxidation and lower P(III) retention.

Our discovery of P(III) and PP_i_ in mafic igneous rocks implies that seafloor weathering could have supplied reactive P to the deep ocean on the early Earth, with important implications for the origin of life on Earth and other habitable worlds and for the bioavailability of P in ancient ecosystems. To estimate the concentration of P(III) in the prebiotic ocean resulting from this flux, we constructed a simple box model in which seafloor weathering was the sole source. Sinks include photochemical oxidation^45^ and dark oxidation (dark oxidation refers to natural oxidation of P(III) in the absence of UV radiation) of P(III) to P_i_ (Fig. 3A; see Methods for details)^7^. We did not consider adsorption as a sink of P(III) because earlier studies noted that P(III) adsorption onto ferrihydrite is limited^11^. Adsorption of P(III) onto other secondary minerals, including clays and related alteration products, has not yet been constrained, but considering that P_i_ adsorption on clays is lower or similar^46^ to P_i_ adsorption on ferrihydrite, we assume that P(III) adsorption on clays is also minor, similar to P(III) adsorption on Fe-oxides. The seafloor weathering flux of P(III) was derived from the corresponding P_i_ weathering flux proposed by Syverson et al.^47^, scaled by the crustal P(III)/P_i_ ratio measured in this study (Table 1). Because CO_2_ uptake by mafic rocks is proportional to P release^47^, the global seafloor P weathering flux is tied to volcanic CO_2_ outgassing. The photochemical sink was assumed to equal the flux of P(III) from the deep ocean to the surface, where rapid photo-oxidation reduces surface concentrations effectively to zero^45^. The abiotic dark oxidation sink was calculated from the half-life of 600,000 years estimated by Herschy et al.^7^. Mineral precipitation was neglected due to the high solubility of P(III) relative to P_i_^7^. A similar model could not be constructed for PP_i_ because its solubility and sinks in aqueous systems are not well constrained.

**Fig. 4:**
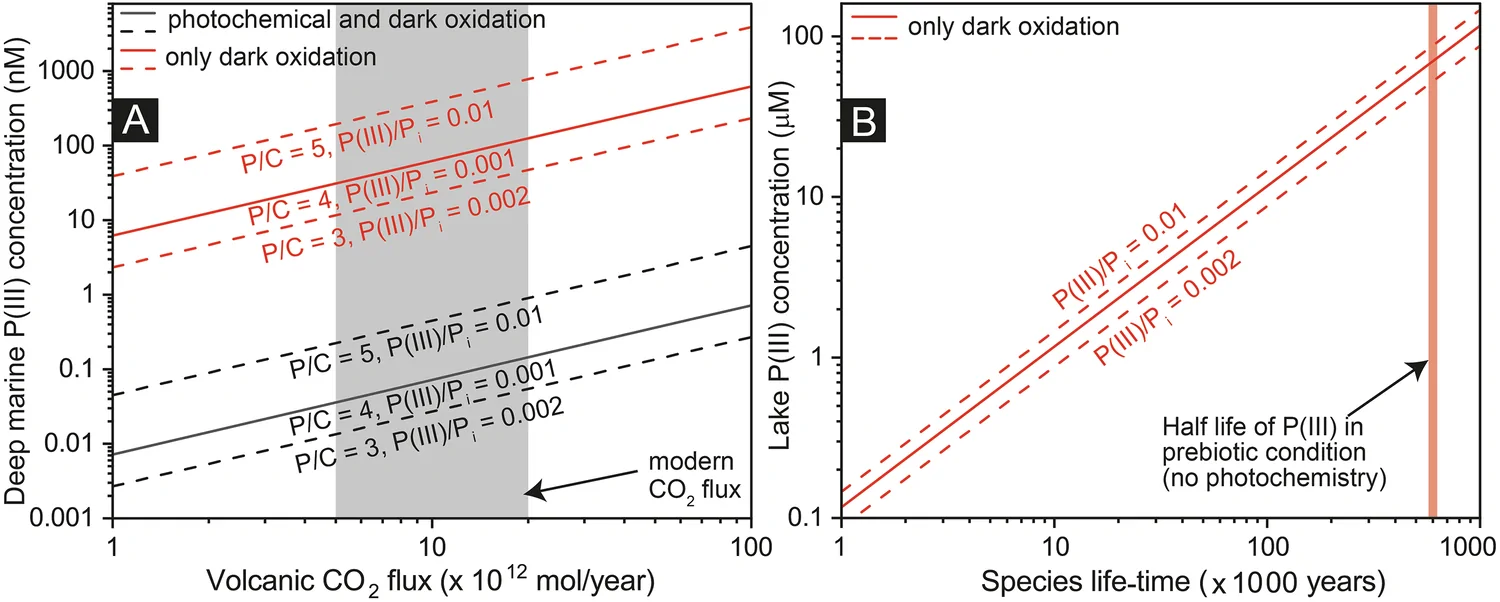
Estimated concentrations of phosphite (P(III)) in early ocean and prebiotic lake environments. **A** Modelled P(III) concentrations in the deep ocean as a function of volcanic CO₂ flux. Estimates incorporate two oxidative scenarios: combined photochemical and dark oxidation (red line) and dark oxidation only (black line). Upper-bound values assume a P-to-CO₂ flux ratio (R_PC_) of 5 (based on the highest experimental value^47^) and the maximum P(III)/P_i_ ratio of 0.01 observed in the studied samples. Lower-bound estimates are based on an R_PC_ of 3 and a P(III)/P_i_ ratio of 0.001. Modern CO_2_ flux are from Syverson et al^47^. Given the elevated volcanic activity on the early Earth^28,29^, a CO₂ flux of 100 × 10^12 ^mol yr^−^^1^ is considered the most realistic. **B** Estimated P(III) concentrations in a prebiotic Mono Lake analogue as a function of P(III) half-life. Upper and lower bounds correspond to the highest and lowest measured P(III)/P_i_ ratios in the studied samples. The longevity of P(III) is strongly influenced by UV exposure: the highest concentrations occur under UV-shielded conditions, where photochemical oxidation is suppressed, and P(III) half-life is extended to that in dark-oxidation-only scenarios (0.6 Ma)^7^.

The model suggests that the prebiotic deep ocean could have maintained an upper-bound steady-state reservoir of up to 1 nM P(III) in a scenario with high CO_2_ outgassing rates and therefore intense seafloor weathering (Fig. 4A). Under conditions that suppress photo-oxidation, such as attenuation at shallower depth (<10 m), an atmospheric haze, or due to the presence of dissolved Fe^2+^,^48,49^ steady-state concentrations could have increased up to 1–2 µM.

In the prebiotic lake analogue, drainage through mafic or ultramafic source and catchment would have supplied the dominant P(III) flux, with both photochemical and dark oxidation acting as sinks (Fig. 3B–D). The lake inventory is highly sensitive to the oxidative half-life of P(III), which depends on UV penetration. Under strong photo-oxidation (half-life <1 year), steady-state concentrations would have remained below 0.1 µM (Fig. 4B). However, under restricted UV exposure—due to organic surface layers^50^ or density stratification^4^—steady-state concentrations could have been much higher, reaching up to 67 µM (Fig. 4B).

### Implications for the origin of life and planetary habitability

This study raises several important implications for the origin of life and planetary habitability. Beyond Earth, our results suggest that magmatic P(III) may occur in the crusts of other planetary and asteroidal bodies. On Mars, Enceladus, and Europa, interactions between water and mafic–ultramafic rocks may have created environments that some studies have proposed as potential cradles for life’s origin^51,52^. Meteorite-derived phosphides may have provided reactive P on Mars^18,45^, but no comparable source has yet been identified for Enceladus or Europa. Furthermore, phosphide content varies widely among meteorite classes, with carbonaceous and ordinary chondrites containing only minor proportions of total P as phosphide (<1% and <10%, respectively)^18,45^. Our data instead point to mafic and ultramafic surface rocks on Mars as a significant reservoir, capable of releasing P(III) and PP_i_ during water–rock interaction. The chondritic rocky cores of Enceladus and Europa^53,54^ may likewise host substantial reduced P, potentially up to ~24% of total P (as observed in some peridotite samples), which could be mobilised into their subsurface oceans via water–rock interaction^55,56^. As the oceans of these moons are shielded from ultraviolet radiation by overlying ice, P(III) may accumulate to higher concentrations than on early Earth, where photochemical oxidation would have limited its stability. While P_i_ has already been detected in Enceladus’ ocean^57^, we suggest that P(III) is also likely present and could have supported prebiotic phosphorylation reactions.

On Earth, our findings contrast with the prevailing view that nearly all P outside the core exists in fully oxidised form^24^. Instead, a reduced reservoir has probably persisted since planetary accretion, supplying reactive species to surface environments. This implies a much larger endogenous supply of reactive P to prebiotic chemistry than previously assumed.

Mafic and ultramafic rocks could also have provided reactive P to prebiotic hydrothermal systems, oceans, and lakes. Fluids from hydrothermal vents—long regarded as plausible sites for life’s emergence^58^—may have contained both P(III) and PP_i_ derived from rock–fluid interaction. Previous work has shown that P_i_ can form high-energy PP_i_ compounds via reactions with acetyl phosphate in Fe-sulfide/silicate precipitates^59^; our results suggest an additional pathway for PP_i_ delivery that would increase the availability of energy-rich polyphosphates in vent systems. In lacustrine settings, P(III) concentrations may have reached tens of micromolar under UV-dark conditions maintained by Fe^2+^,^48^ an organic surface layer,^50^ or stratification^4^, particularly if the supply was stable. Although experimental constraints on the concentrations of P(III) required for phosphorylation are limited^6,10^, a komatiitic source with a higher P(III)/P_i_ ratio coupled with wet–dry cycles that enhance solute accumulation and the greater reactivity of P(III) relative to P_i_, suggests that such concentrations could have been sufficient to drive prebiotic phosphorylation chemistry.

In the ocean, reactive P released from mafic crust may have constituted a stable, long-term source of P during the Archaean. The availability of P_i_ in Archaean seawater remains debated, with some studies arguing for high P_i_ abundance^60–62^ and others suggesting strong P-limitation^63–65^. During this time, meteorites have likely been an important source of P(III), owing to its greater solubility^12^. Our results indicate that in the absence of extraterrestrial or biological inputs, and under strong UV irradiation, the concentration of P(III) derived from seafloor weathering was likely low. This may help explain the relatively late emergence of P(III)-utilising enzymes near the Archaean–Proterozoic boundary^66^. Nonetheless, the high solubility of P(III) and its photochemical oxidation would have provided an indirect flux of P_i_ to the early biosphere. Recent estimates indicate that during the Great Oxidation Event, P(III) accounted for 5–88% of dissolved inorganic P, at concentrations up to 0.17 μM^11^. In light of our data, this reservoir may have been at least in part sustained by crustal weathering under reduced UV flux or augmented by early biological inputs of reduced P.

Altogether, our findings show that mafic and ultramafic rocks could have been an important reservoir of reactive P on the early Earth. Up to ~0.70% of total crustal P may have occurred as P(III) and PP_i_ in the Archaean, yielding upper-bound P(III) oceanic concentrations of hundreds of nanomolar and lake concentrations of tens of micromolar. Transiently elevated levels of these reactive P species could have fuelled prebiotic phosphorylation while maintaining a stable, long-term P source in the Archaean ocean. Comparable P(III) reservoirs on Mars, Europa, and Enceladus may likewise have expanded the availability of reactive P and the potential for life.

## Methods

### Quantification of phosphorus species in rock samples

All glass and plastic containers used in this study were cleaned with 1 M HCl followed by two rinses with hot water and dried. Glass containers were subsequently baked at 500 °C to ensure complete removal of organic contaminants. Rock and mineral samples were initially crushed into millimetre-sized chips, then sequentially washed with methanol and 1 M HCl (four times each) to eliminate surface contaminants. After rinsing five times with deionized (DI) water, the samples were freeze-dried and further pulverized using a ball mill.

For phosphorus (P) extraction, an aliquot (ca. 0.2–0.25 g) of the powdered sample was treated with an ethylenediaminetetraacetic acid-sodium hydroxide (EDTA-NaOH) solution (0.05 M EDTA and 0.25 M NaOH) at a solid-to-solution ratio of 1:10 for 14–15 hours at room temperature on a rotary shaker. The EDTA-NaOH solution was prepared by dissolving Na₂EDTA (Sigma-Aldrich) and 10 M NaOH (Thermo Scientific) in DI water. The extraction was conducted in 15 mL Falcon tubes. Following extraction, the solutions were centrifuged at 3000 rpm for 15–20 minutes, and the clear supernatant was collected for analysis.

Four P species, namely hypophosphite, phosphite, phosphate, and pyrophosphate, were analyzed using an ion chromatography-inductively coupled plasma mass spectrometry (IC-ICP-MS) system^25^. The IC system (Thermo Scientific Dionex ICS-6000) was equipped with a Dionex AS-AP autosampler, an IonPac AS17-C separation column (250 mm, 2 mm bore), an IonPac AG17-G guard column (50 mm, 2 mm bore), and an ADRS-600 suppressor (2 mm). Prior to analysis, the extracted supernatant was diluted 25-fold to prevent column interference from high metal and EDTA concentrations.

Chromatographic separation was achieved using a KOH eluent gradient (1–40 mM over 20 min), maintained at 40 mM for additional 25 min, then ramped back to 1 mM over next 5 min, with a constant flow rate of 0.5 mL/min. The IC suppressor outlet was directly coupled to a Scott-type quartz spray chamber via a 1 mL/min nebulizer, connected to an Element 2 ICP-MS. The suppressor was regenerated with deionized water at 1 mL/min using an external pump. The ICP-MS was operated in medium resolution mode with the following parameters: RF power, 1183 W; cool gas flow, 16 L/min; and sample gas flow, 1.1 L/min. Data acquisition involved 3-minute chromatograms per P species, comprising 1 min of pre-peak background, 1 min of peak monitoring, and 1 min of post-peak background measurements.

Chromatographic data were smoothed using OriginLab software with a Fast Fourier Transform (FFT) filter (window size = 5 points), and peak areas were used for P quantification. Calibration standards for all four P species—prepared from NaH₂PO₂·H₂O (hypophosphite), Na₂HPO₃·5H₂O (P(III)), NaH₂PO₄ (P_i_), and Na₄P₂O₇ (PP_i_)—were matrix-matched to the sample EDTA-NaOH solution and covered a concentration range of 0.2–100 ppb. These standards were analyzed identically to the samples, and their peak integrals were used to construct calibration curves for quantification. The starting reagents (HCl, EDTA-NaOH, DI water) were analyzed as procedural blanks, and it is noted that none of these reagents contain P(III) (Supplementary Fig. S2). Furthermore, we analyzed a negative control, silica powder, which was heated to 500 °C in air overnight (to oxidize all P(III), if present, into Pi). P(III) was not detected in the EDTa-NaOH extract (Supplementary Fig. S2). Blanks prepared with EDTA–NaOH but without samples reveal minor levels of P(V) and PP_i_, which were corrected for the samples (Supplementary Fig. S2). Method detection limits (MDLs) in the solutions were determined to be <0.1 ppb for P(III) and P_i_, 0.1 ppb for hypophosphite, and 0.2 ppb for PP_i_.

### Whole rock analysis

Approximately 0.30–0.60 g of powder from each of the samples was sent to Australian Laboratory Services (ALS) in Dublin, Ireland, for whole-rock geochemical characterisation using their method ME-MS-61r of four-acid digestion (HCl, HNO_3_, HF, HClO_4_) followed by ICP-MS and -AES analyses. Reproducibility was assessed with rock standards OREAS-45d (50:50 blend of mineralised lateritic soil and barren soil), OREAS-905 (a blend of copper oxide ore and rhyodacite), and MRGeo-08, and with two or three sample replicates. It was found to be 5 % or better for P and trace elements. Whole rock data is provided as Supplementary Data 2.

### Estimation of oceanic crustal reactive phosphorus reservoir

To estimate the temporal evolution of oceanic crustal reactive P, we considered the compositional change of oceanic crust over geological time. During the Archaean, komatiite and basalt were the dominant lithologies forming the oceanic crust, although komatiite generation declined significantly after 2.5 Ga. Following the approach of Greber et al.^44^, who used titanium isotope systematics to infer the proportions of major lithologies in the continental crust from 3.5 to 2.0 Ga, we adopted analogous ratios for the oceanic crust, assuming it was composed solely of basalt and komatiite. For instance, at 3.5 Ga, the oceanic crust is modelled to consist of 64% basalt and 36% komatiite, based on the estimated 27% basalt and 15% komatiite contributions to the contemporaneous continental crust^44^. Using these proportions, together with our constrained average concentrations of P(III) and PP_i_ in basalt and komatiite, we quantified the evolving abundance of reactive P species in the oceanic crust from 3.5 to 2.0 Ga.

### Estimates of reactive phosphorus in the prebiotic ocean and lakes

The crustal estimation of P(III) enables us to quantify its source flux into the early Earth’s ocean by seafloor weathering. On the other hand, previous studies have identified potential sinks for P(III) in the early Earth’s oceans^7,45^. These source and sink fluxes were incorporated into a box model to estimate the concentration of P(III) in the early Earth’s deep ocean. Syverson et al. (2021)^47^ demonstrated that aqueous alteration of mafic crust under anoxic conditions can liberate P_i_ into seawater through reaction with carbonic acid. Based on laboratory experiments, they derived an empirical P_i_-to-CO₂ release ratio (R_PC_) of 4 ± 1 µmol/mmol. This ratio was used to estimate the global P_i_ weathering flux by multiplying R_PC_ with the global volcanic CO₂ outgassing flux (*J*_*volc*_) and the fraction of CO₂ consumed during reaction with mafic crust (*f*_*wx*_).

Building on this framework, we extended the P_i_ weathering flux to estimate the P(III) weathering flux, incorporating our measured P(III)-to- P_i_ ratio (P(III)/ P_i_) in mafic igneous rocks. The resulting P(III) input to the prebiotic ocean (*P*_*hyd*_) is given by:

1 $${P}_{{hyd}}={J}_{{volc}}\cdot {f}_{{wx}}\cdot \frac{P\left({III}\right)}{{P}_{i}}$$

Reported values for *J*_*volc*_ on modern Earth range from 5 to 20 × 10^12 ^mol/yr^47^. We explored a broader range of 1–100 × 10^12 ^mol/yr to account for the possibility of enhanced volcanic activity on the early Earth^67,68^. Syverson et al.^47^ varied *f*_*wx*_ between 0.1 and 0.9 to represent a range of Archaean conditions; in our model, we fixed *f*_*wx*_ at 0.9, assuming an ocean-dominated prebiotic Earth^69^ where seafloor alteration acted as the primary carbon sink. The P(III)/P_i_ ratio was varied from 0.001 to 0.01, with an average value of 0.002 based on our measurements.

The main sinks of P(III) are linked with its photochemical^45^ and dark^7^ oxidation to P_i_, among which photochemical oxidation dominates. Ritson et al.^45^ demonstrated that of P(III) is rapidly oxidized to of P_i_ under experimental ultraviolet irradiation, with complete conversion occurring within hours, especially in the presence of H_2_S/HS^–^. In naturally relevant UV conditions, photochemical oxidation may be slow, on the order of tens of years^45^. However, this slow time is still similar to the residence time of water in the ocean’s photic zone (which is ca. 28 years, assuming a surface ocean volume (*V*_*photic*_) of 3.62·10^16^ m^3^ and a downwelling flux (*F*_*down*_) of 1.26·10^15^ m^3^/yr, so that the residence time is *V*_*photic*_*/F*_*down*_ = 28 years, after Emerson & Hedges^70^. We therefore assumed that all P(III) transported to the surface ocean was instantaneously oxidized. The photochemical sink (*P*_*photo*_) was calculated as:

2 $${P}_{{photo}}={F}_{{mix}}\cdot {P({III})}_{{deep}}$$

where *F*_*mix*_, the global marine upwelling flux, was fixed at 10^15^ m^3^/yr^71^, and *[P(III)]*_*deep*_ is the deep ocean P(III) concentration, calculated by numerical integration (see below).

To account for dark (non-photochemical) oxidation of P(III), we used a half-life (τ) of 600,000 years under Archaean conditions, as reported by Herschy et al.^7^ This half-life value is valid for ferruginous anoxic conditions and thus particularly relevant to the early Earth ocean. In more recent time periods, after the Great Oxidation Event, P(III) oxidation would also be impacted by molecular oxygen and biology. However, these parameters were not considered here as our model focuses on the prebiotic ocean. The associated first-order rate constant (*k*_*ox*_) was calculated as: *k*_*ox*_ = ln(2)/τ. The total dark oxidation sink (*P*_*dark_ox*_) was then:

3 $${P}_{{dark}{{\_}}{ox}}={K}_{{ox}}\cdot {V}_{{deep}}\cdot {P({III})}_{{deep}}$$

where V_deep_, the volume of the deep ocean^72^, was set to 10^18^ m^3^.

The overall mass balance for the deep ocean P(III) reservoir, i.e., the rate of change of P(III) accumulation in deep ocean, can be described by the following ordinary differential equation:

4 $$\frac{{dP}}{{dt}}=\frac{\left({P}_{{hyd}}-\,{P}_{{photo}}\,-{P}_{{dark}{{\_}}{ox}}\right)\,}{{V}_{{deep}}}$$

or explicitly:

5 $$\frac{{dP}}{{dt}}=\frac{\left(\left({J}_{{volc}} * {R}_{{PC}} * {f}_{{wx}} * \frac{P({III})}{{P}_{i}}\right)-\,\left({F}_{{mix}} * {P({III})}_{{deep}}\right)\,-\left({k}_{{ox}} * {V}_{{deep}} * {P({III})}_{{deep}}\right)\right)\,}{{V}_{{deep}}}$$

This differential equation was numerically solved using the *odeint* solver in Python (Supplementary Data 2). The model was run until steady state was reached, and the resulting equilibrium concentration of *[P(III)]*_*deep*_ was recorded and plotted. The code developed to solve this equation is given in the Supplementary material.

To quantify steady-state P(III) concentrations, we modelled a hydrothermally fed soda lake under closed-basin conditions, where hydrologic inputs are fully balanced by evaporation. In such systems, dissolved species approach equilibrium when removal by oxidative sinks (e.g., photolysis, hydrolysis, or precipitation) offsets influx. The steady-state concentration C of a solute can be expressed by:

6 $${C}_{i}=\,\frac{{F}_{i} * {L}_{i}}{V}$$

Where *C*_*i*_ is the concentration (mol/L) of a species *i*, *F*_*i*_ is the flux (mol/year) of species *i* entering the lake system, *L*_*i*_ is the lifetime (year) of species *i* in the lake environment, and *V* is volume (litre) of water in the lake.

We considered the accumulation of P(III) in the hypothetical lake, exploring lifetime as a free parameter and then highlighting the expected value for anoxic early Earth systems based on available experimental evidence. For values of flux and volume, we considered a hydrothermally active soda lake with similar hydrological properties to Mono Lake in California^4^. In other words, the hypothetical lake is a prebiotic analogue of modern Mono Lake. For Mono Lake, the volume of the lake *V* = 3 * 10^12^ litre and P_i_ inflow rate *F*_*Pi*_ is = 1 × 10^−3^ mmol/year^4^. With the observed ratios of P(III)/P_i_ in the basalt samples, we have considered three phosphite annual inflow rate (*F*_*P(III)*_*)* values - 0.001 µmol/year, 0.01 µmol/year, and 0.1 µmol/year. We have considered two endmember cases in terms of the extent of photochemical oxidation in these types of lake. If the lake was exposed to UV-radiation, phosphite oxidation would have been rapid, leading to sub-nanomolar or lower concentrations of P(III). On the other extreme end, we assumed that photochemistry is not occurring, requiring efficient UV attenuation in the lake surface. This may be accomplished by the accumulation of effective UV-absorbing organics in surficial ‘scum’ layers^50^ or by the density segregation of the lake to form basal dark layers^4^.

## Supplementary information

Supplementary Information

Description of Additional Supplementary Files

Supplementary Data 1

Supplementary Data 2

Transparent Peer Review file

## Data Availability

All data are available in the main text or in the supplementary materials. Phosphorus speciation data can also be accessed from British Geological Survey at 10.5285/18ee8cec-f141-4544-b210-aa3a4f0a557b (details: Baidya, A. S., Walton, C. R., Kalita, J., Szilas, K., Viccaro, M., Savage, P., Allison, S., Nisbet, E., Schönbächler, M., Mikhail, S., Stüeken, E. E. (2025). Phosphorus speciation and metal abundances in mafic and ultramafic rocks. NERC EDS National Geoscience Data Centre. (Dataset)).

## Code availability

The Code developed for phosphite modelling is provided as Supplementary Data.
