# Supplementary material for: Mafic-ultramafic igneous rocks as a source of reactive phosphorus for the origin of life
Source: Nat Commun. 2026 Jul 23;17:8994. doi: 10.1038/s41467-026-75933-6 (PMC13507249; doi:10.1038/s41467-026-75933-6)
Supplement: Supplementary file 1 [file 41467_2026_75933_MOESM1_ESM.pdf]

Mafic-ultramafic igneous rocks as a source of reactive phosphorus for the origin of life

Abu Saeed Baidya[1], Craig R. Walton[2,3], Joanna Kalita[1], Kristoffer Szilas[4], Marco Viccaro[5,6], Paul S. Savage[1], Stuart Allison[1], Euan Nisbet[7], Maria Schönbächler[2], Sami Mikhail[1], Eva E. Stüeken[1]

[1]School of Earth and Environmental Sciences, University of St. Andrews, St Andrews KY16 9TS, United Kingdom

[2]Department of Earth and Planetary Sciences, ETH Zurich, Sonneggstrasse 5, 8092 Zurich, Switzerland

[3]Trinity College, University of Cambridge, Cambridge CB2 1TQ, United Kingdom

[4]Natural History Museum of Denmark, University of Copenhagen, Øster Voldgade 5-7, 1350 Copenhagen, Denmark

[5]Department of Biological, Geological and Environmental Sciences, University of Catania, Italy

[6]National Institute of Geophysics and Volcanology, Section of Catania – Ossevratorio Etneo, Italy

[7]Department of Earth Sciences, Royal Holloway, University of London, Egham TW20 0EX, UK

Corresponding Author: Abu Saeed Baidya (asb27@st-andrews.ac.uk)

This File contains:

- Supplementary Notes
- Supplementary Figures with captions
- Supplementary Table 1
- Supplementary References

Supplementary Notes: Sample description

Basalt samples (n = 6) were collected from the Canary Islands (n = 1), Spain (n = 1), Kilbourne Hole, USA (n = 2), and Mount Etna, Italy (n = 1). In addition, previously published data from basalts from the Moodies Group, South Africa (n = 2), were incorporated[1] (Supplementary Table 1, Figure S1 in Supplementary Material).

Overall, major- and trace-element compositions overlap with those of ocean-island basalts (OIB) and mid-ocean-ridge basalts (MORB) in MgO–Ni, MgO–CaO and MgO–P_2O_5 space (Fig. S1a–c). Consistent with this, previous studies report that basalts from the Canary Islands and Kilbourne Hole are OIB-like[2,3]. A positive U–K_2O correlation indicates minimal alteration (Fig. S1d). We therefore consider these basalts as representative of early-Earth ocean-floor lavas and use them in models estimating phosphite concentrations in ocean and prebiotic-lake settings.

Olivine samples (n = 4) comprise one from a pallasite meteorite, one fayalite from the Lasigkulla mine (Sweden), one from lherzolite, and one from Hawaiian basalt. Among them, the last two are considered as ‘olivine separates’. The fayalite sample may contain minor accessory phases. Regarding peridotite samples (n = 15), one is harrisite from the Isle of Rum (Scotland), one is harzburgite, and the rest are dunite (Supplementary Table 1). The harrisite specimen shows intergrowths of olivine and plagioclase. These rocks show no petrographic or geochemical evidence of alteration, and their CaO and Al_2O_3 contents are consistent with magmatic differentiation (Supplementary Fig. S3).

Komatiite samples were collected from the Belingwe greenstone belt, Zimbabwe, and show no or minor alteration[4].

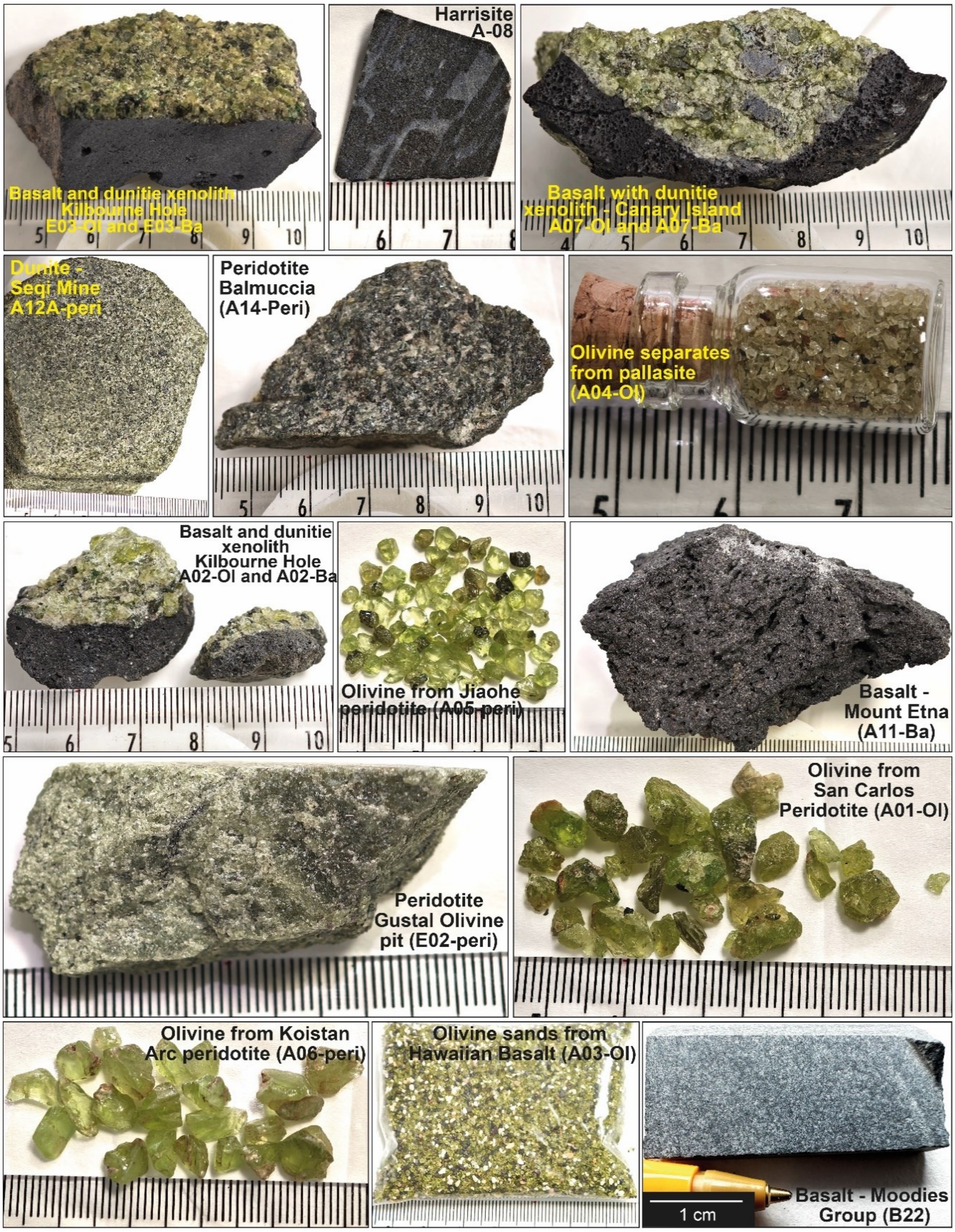

Figure S1: Photographs of selected samples used in this study. *Most of the samples do not have any indication of alteration. Top panel from left: basalt and dunitic xenolith from Kinbourne Hole, harrisite from Rhum Islan, and basalt and dunitic xenolith from Canary Island. 2nd panel from left: dunite from Seqi Mine, peridotite from Balmuccia, and olivine separate from pallasite meteorite. 3rd panel from left: basalt and dunitic xenolith from Kilbourne Hole, olivine separates from Jiaohe peridotite, and volcanics from Mount Etna. 4th panel from left: peridotite sample from Gustal olivine pit and olivine separates from San Carlos peridotite. Bottom panel from left: olivine separates from Kohistan Arc, olivine sand*

from Hawaii, and basalt sample from Moodies group. For detailed sample description, readers are referred to Supplementary Table S1.

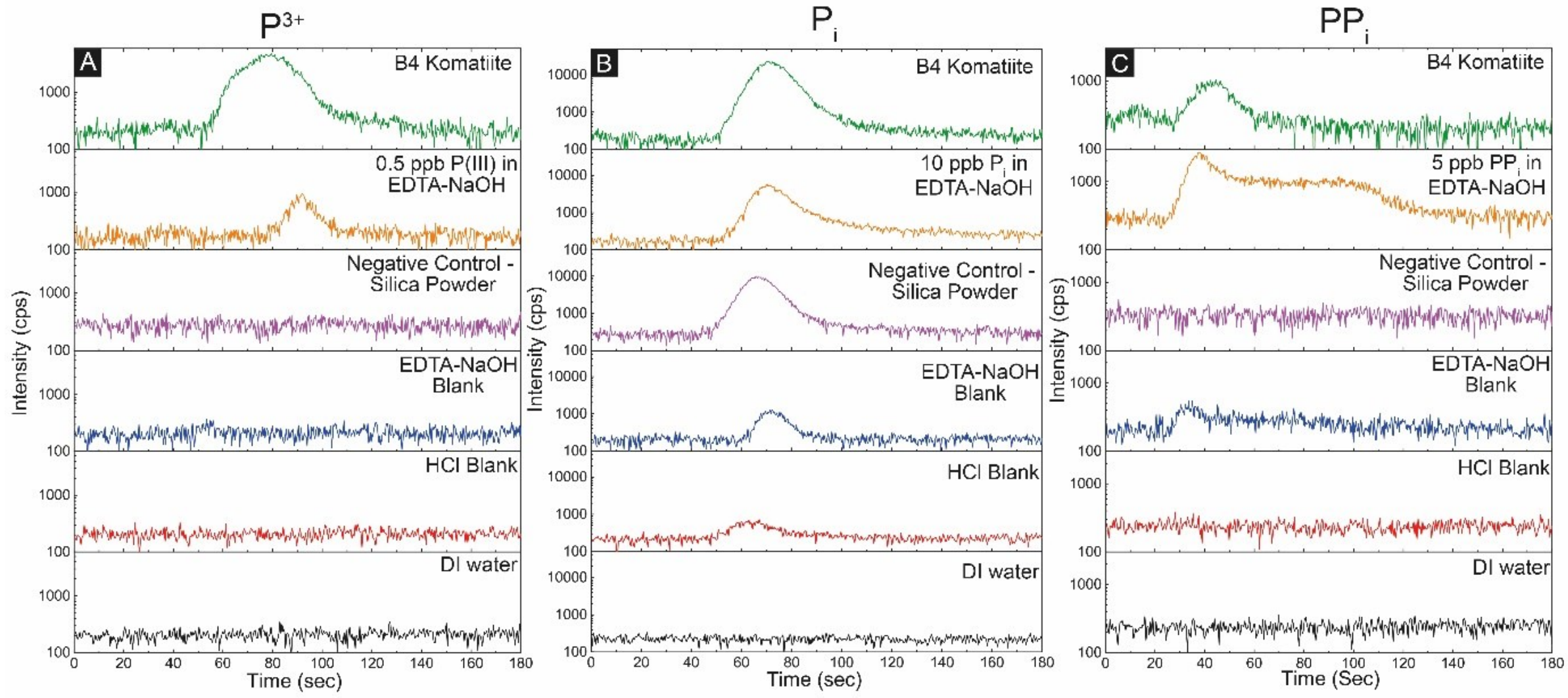

***Figure S2: IC–ICP–MS chromatograms of P(III), Pi, and PPi in starting materials, blanks and selected samples.** A, No detectable P(III) is observed in DI water, washing HCl or the EDTA–NaOH extractant. Silica powder, prepared from powdered sea sand baked at 500 °C in air to oxidize any pre-existing P(III), also shows no detectable phosphite. By contrast, the 0.5 ppb P(III) standard and a representative komatiite sample show clear phosphite peaks. B, Pi is detected in all starting materials except DI water; blank contributions from EDTA–NaOH were subtracted from sample values. C, No detectable PPi is observed in DI water or HCl, whereas EDTA–NaOH contains a minor PPi blank that was corrected for in the samples.*

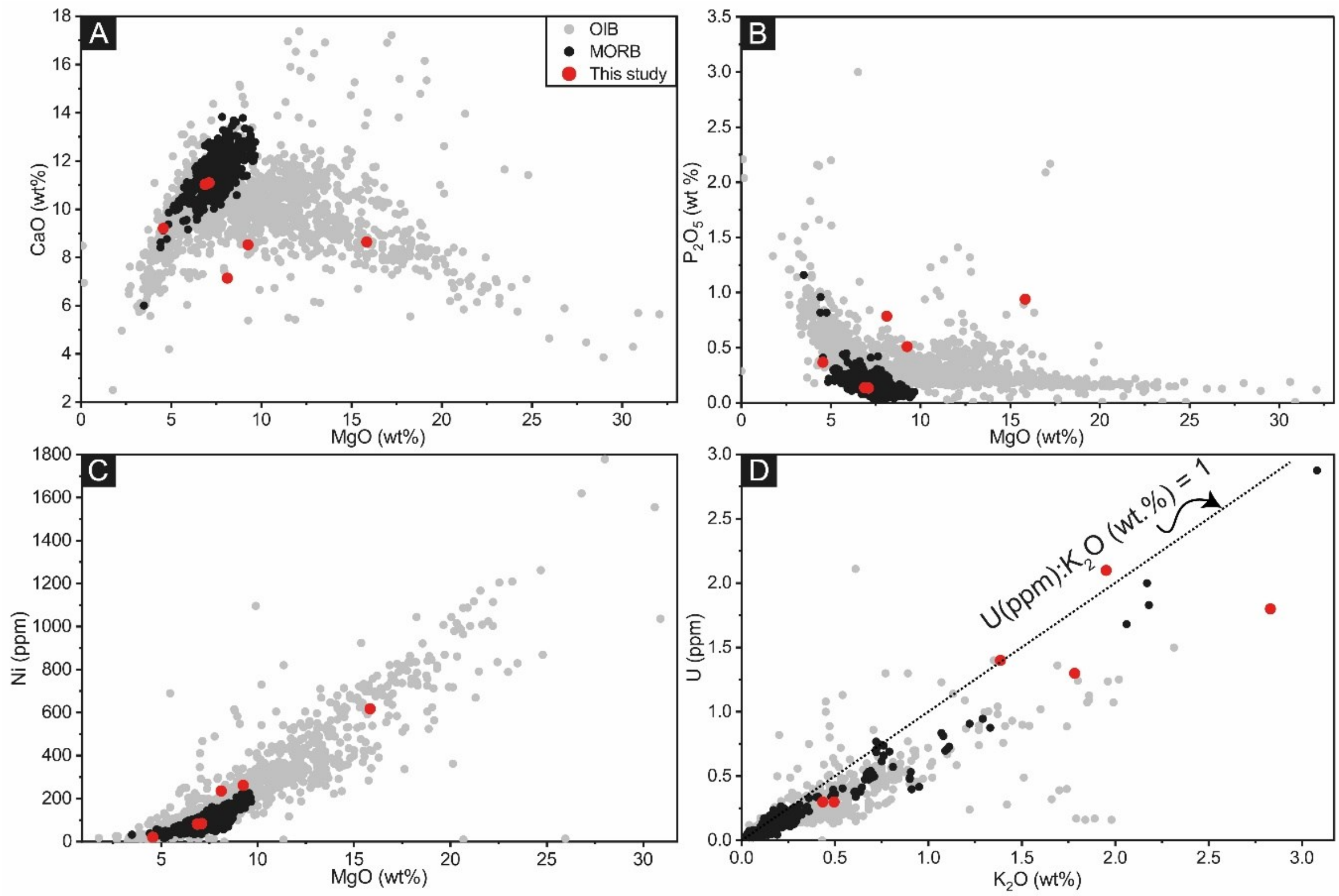

Figure S3: Comparison of the studied basalts with ocean-island basalts (OIB) and mid-ocean-ridge basalts (MORB). *MORB and OIB data are from Stracke et al.*[5] *(A) MgO versus CaO; (B) MgO versus P_2O_5; and (C) MgO versus Ni. In A–C, samples span both OIB and MORB compositional fields. (D) K_2O and U are positively correlated, indicating minimal alteration.*

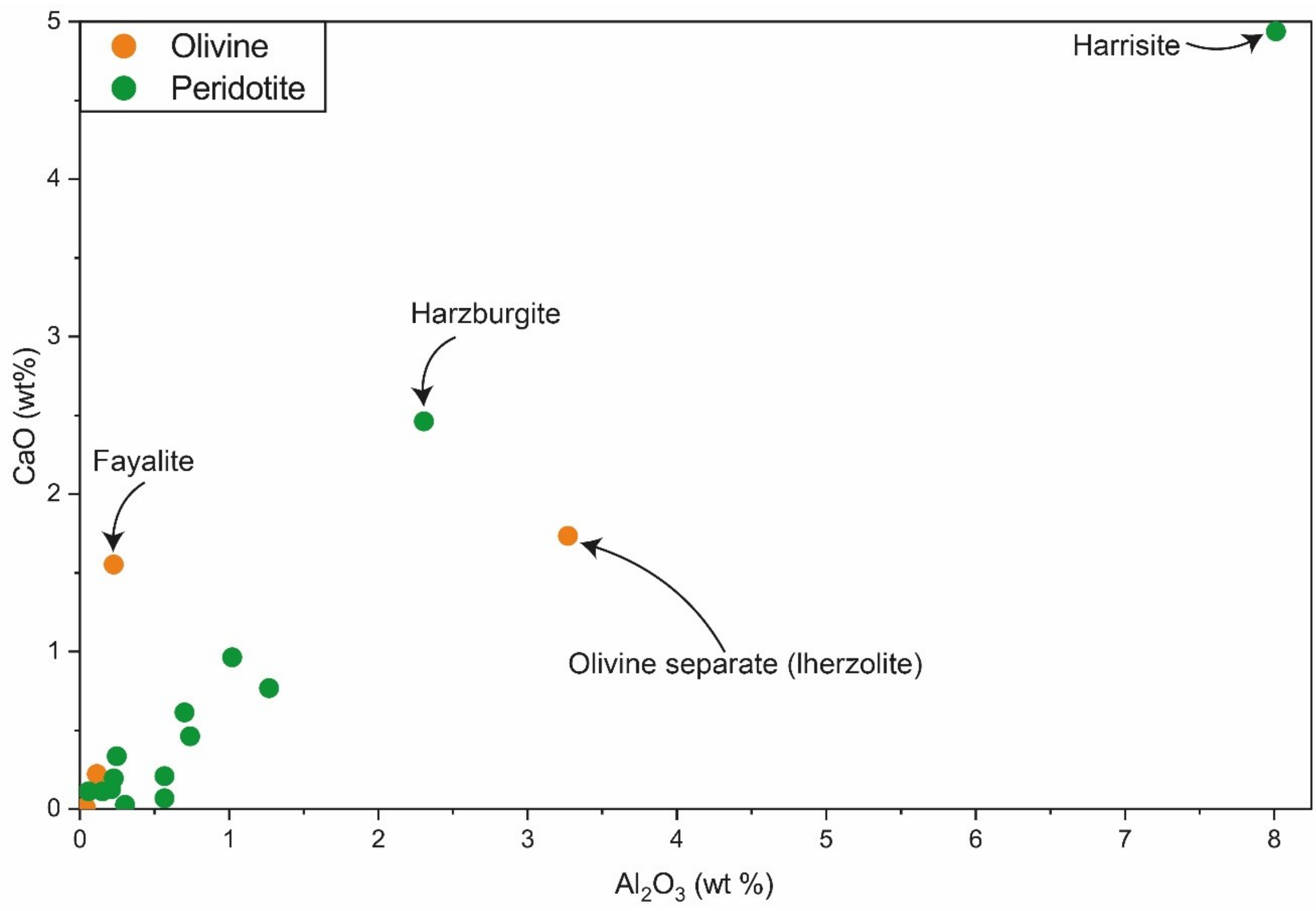

Figure S4: Olivine and peridotite samples show a positive correlation between calcium oxide (CaO) and aluminium oxide (Al_2O_3). *This trend suggests magmatic differentiation. Harrisite is an intergrowth of olivine and plagioclase while fayalite sample contain some unidentified phases, which may explain relatively higher Al and/or Ca contents in these samples. The olivine separates from lherzolite may contain some pyroxene grains or inclusion, which explains relatively higher Al and Ca contents.*

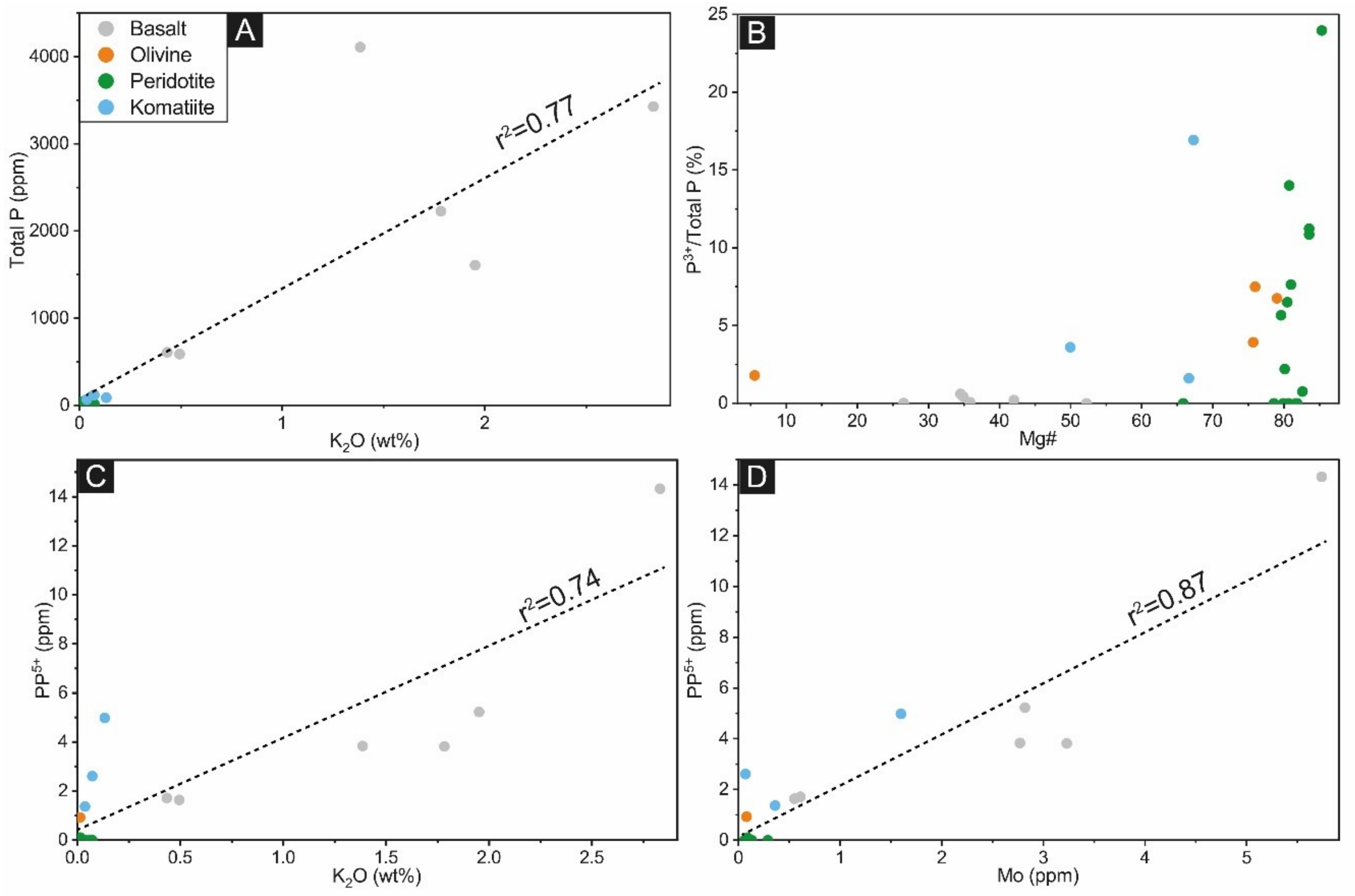

Figure S5: Phosphorus chemistry and its correlations with major and trace elements in the studied samples. *(A) Phosphorus (P) versus potassium oxide (K_2O) shows a positive correlation consistent with co-enrichment during magmatic differentiation. (B) Samples with higher magnesium number (Mg#) exhibit greater phosphite [P(III)] to total phosphorus (ΣP) percentages. (C–D) Pyrophosphate (PP_i) correlates positively with potassium oxide (K_2O) and with molybdenum (Mo), respectively.*

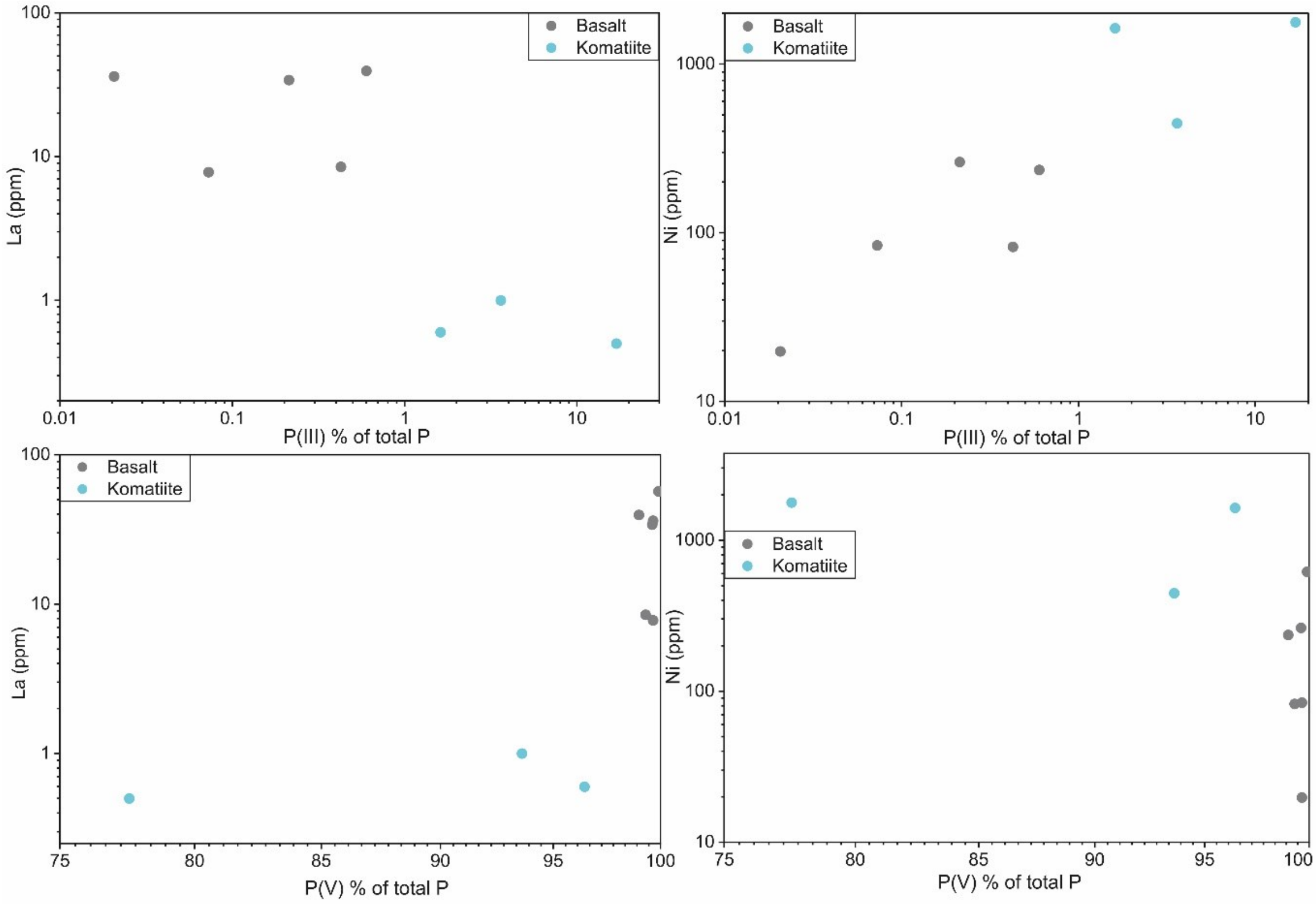

Figure S6: Relationship between magmatic differentiation indicators (La and Ni) and P(III)% and P_i%. *P(III)% has weak negative correlation with La and moderately positive correlation with Ni, whereas P_i% does not show the same relationships. This pattern is consistent with the possibility that P(III) behaves more compatibly than P_i during magmatic differentiation.*

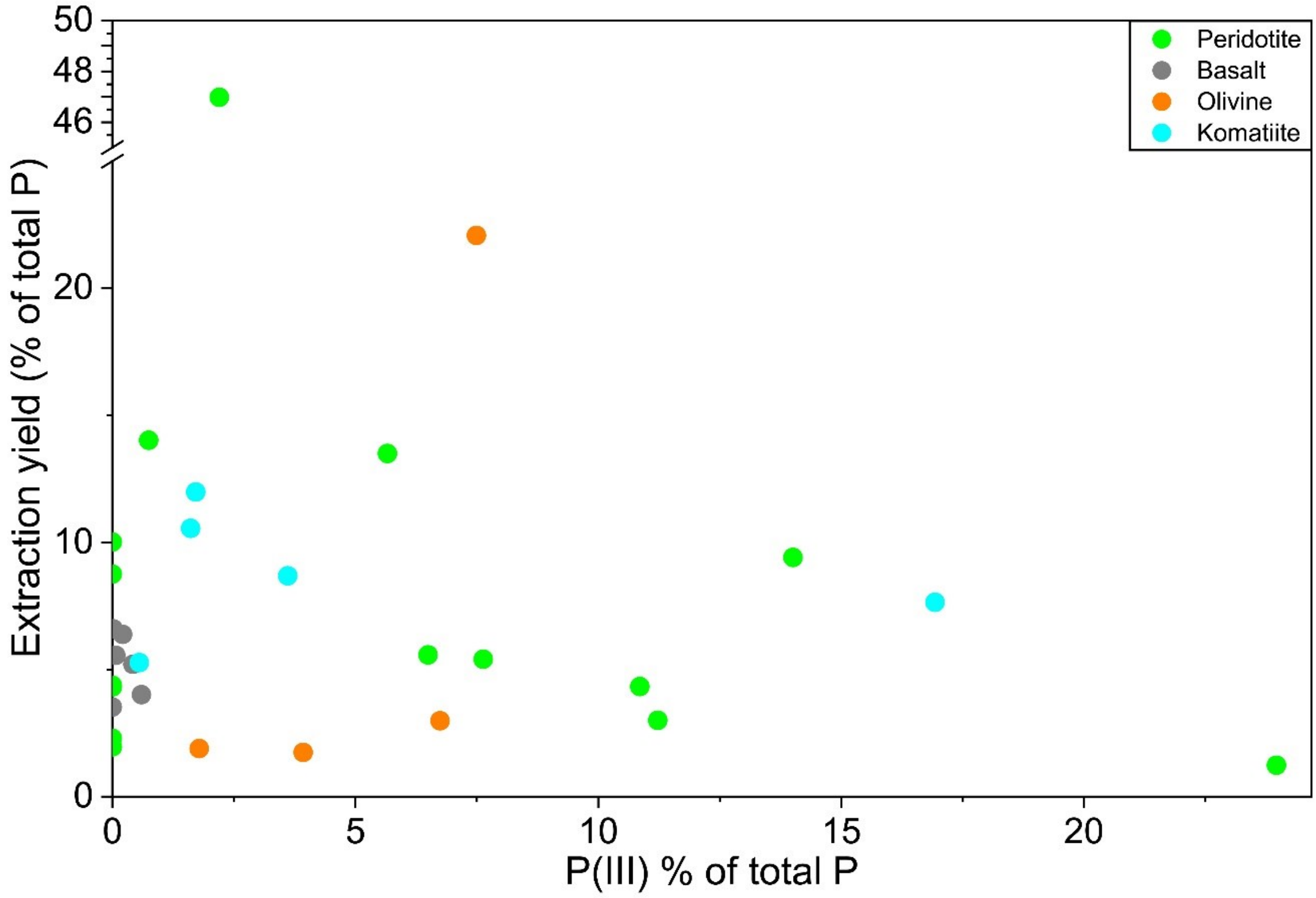

Figure S7: P extraction yields in EDTA-NaOH solution and P(III)/ΣP ratio plot show no correlation. *This indicates there was no preferential extraction of P(III) and P_i.*

Supplementary Table S1- List and description of the samples

Samples	Rock/mineral type	Location	Age, Tectonic Settings, and Sample description
E03-Ba	Basalt	Xenolith host, Kilbourne Hole	These two samples consist of dunitic xenoliths contained in basaltic host rock from Kilbourne Hole, Potrillo volcanic field, New Mexico, USA. Kilbourne Hole is a Quaternary maar within the Rio Grande rift and represents a continental intraplate extensional volcanic setting[6]. The host basalt has been described as basanitic to basaltic and erupted at approximately 10 ka[6]. Basaltic samples were physically separated from the xenolith and analyzed independently from the xenoliths. They do not show any alteration and have compositional characteristic of mid oceanic ridge basalts.
A02-Ba	Basalt	Xenolith Host, Kilbourne Hole	
A07-Ba	Basalt	Xenolith Host, Canary Island	The sample consist of a dunitic xenolith hosted in basaltic lava from the 1730–1736 CE Timanfaya eruption, Lanzarote, Canary Islands[7]. Lanzarote belongs to the Canary volcanic province, an oceanic intraplate volcanic setting related to hotspot magmatism[8]. The basaltic part of the sample is vesicular, unaltered, and has a compositional characteristic of ocean island basalt.
A11-Ba	Basalt	Volcanics, Mount Etna	The sample is a K-trachybasaltic lava erupted on 24 December 2018 from Mt Etna, a volcano in eastern Sicily located in a geodynamically unusual setting at the junction of the Apennine–Maghrebian thrust belt, the Hyblean Foreland, and the Ionian oceanic domain[9,10]. Although Etna sits above a compressive regional tectonic setting, its magmas show a marked intraplate oceanic affinity rather than a typical subduction signature[11]. Petrographically, the lava is porphyritic (25–35 vol.%) and contains plagioclase, clinopyroxene, olivine, and Ti-magnetite as phenocrysts and microphenocrysts in a microlite-rich hyalopilitic groundmass.[12] Plagioclase displays stable core compositions (An 77-85) with more sodic rims (An 66-71), mirroring the chemistry of the groundmass microlites. Clinopyroxene maintains an augitic composition with high Mg# (0.83-0.88). Olivine shows a broad compositional range (Fo 66-87), though cores are predominantly clustered between Fo 75-78.
B22-Ba	Basalt	Moodies Group Intrusive	The sample is an intrusive igneous rock associated with the Paleoarchean Moodies Group of the Barberton Greenstone Belt, South Africa. The Moodies Group is ca. 3.22 Ga in age and forms the uppermost unit of the Barberton Supergroup[13]. The tectonic setting is generally interpreted as a tectonically active Archean basin along the margin of the Barberton Greenstone Belt, with ongoing uplift, sediment supply, and mafic magmatism during Moodies deposition[14]. The sample is taken from the mafic intrusive bodies and contain pyroxene, plagioclase, ilmenite, and olivine. Ilmenite shows skeletal textures and is mostly associated with fine-grained feldspar, pyroxene, glassy material, and rare apatite.[1] Olivine shows minor alteration along grain boundaries and fractures; however, the overall alteration in the rock is limited.
B23-Ba	Basalt		
B4	Komatiite	Belingwe greenstone belt, Zimbabwe	These samples are Neoarchean komatiitic rocks from the Reliance Formation, Ngezi Group, Belingwe Greenstone Belt, Zimbabwe. The Reliance Formation represents the lower ~1 km of a ~6 km thick mafic subaqueous volcanic succession deposited on older tonalitic crust and is now dated at 2746 ± 4 Ma[4,15]. The Belingwe belt is exceptionally well preserved, with low strain, low metamorphic grade, preserved chill zones, and fresh olivine-hosted glass inclusions in some flows[4,16]. ZV10 is an olivine-phenocryst–rich to cumulate komatiite and ZV14 a
ZV 10	Komatiite		
ZV 14	Komatiite		

ZV 15	Komatiite		spinifex-textured komatiite from Tony's Flow, whereas B4, ZV15, and ZV79 are best described more generally as Reliance Formation komatiites unless more specific facies information is available[4,17].
ZV 79	Komatiite		
A08-Ol	Peridotite	Olivine-plagioclase intergrowth, Rhum Island	This sample is harrisite from the Rum Layered Suite, Isle of Rum, NW Scotland[18]. The Rum Central Igneous Complex formed during the Palaeogene at about 60.5 Ma within the British Palaeogene Igneous Province and represents an intraplate mafic–ultramafic intrusive centre related to North Atlantic rifting[19,20]. The sample can be described as an olivine-rich harrisite with probable plagioclase from a layered ultrabasic intrusion.
A07-Ol	Peridotite	Dunite peridotite, Canary Island	This sample consists of a dunitic xenolith hosted in basaltic lava from the 1730–1736 CE Timanfaya eruption, Lanzarote, Canary Islands[7]. Lanzarote belongs to the Canary volcanic province, an oceanic intraplate volcanic setting related to hotspot magmatism[8]. The dunitic part of the sample is unaltered and composed dominantly of forsterite olivine.
E03-Ol	Peridotite	Dunite peridotite, Kilbourne Hole	These two samples consist of dunitic xenolith in basaltic host rock from Kilbourne Hole, New Mexico, USA, as mentioned above. Dunitic peridotite is composed of forsterite olivine (see Supplementary data for composition).
A02-Ol	Peridotite	Dunite peridotite, Kilbourne Hole	
A10-Ol	Peridotite	Dunite peridotite, Ubekendt Island	The sample is a dunite peridotite from Ubekendt Ejland, West Greenland, derived from mantle material carried by alkaline basaltic to basanitic lavas. Ubekendt Ejland belongs to the West Greenland volcanic province within the North Atlantic Igneous Province and records late Palaeogene (53 Ma) continental rift-related / intraplate magmatism associated with North Atlantic opening[21–23]. Mantle xenoliths, including dunite and lherzolite, are documented from Ubekendt Ejland lavas, and dunitic xenoliths have been described specifically from late Palaeogene alkaline basalt flows. The olivine is derived from dunite xenoliths entrained by the ascending host magma and forsteritic in composition. Olivine grains do not show any alteration.
E01-Ol	Peridotite	Dunite peridotite, San Carlos, Arizona	These two samples are dunitic peridotite from the San Carlos, Arizona. See below for tectonic settings and age information for San Carlos volcanics.
A09-Ol	Peridotite	Peridotite, San Carlos, Arizona	
A01-Ol	Peridotite	Dunite peridotite, San Carlos, Arizona	The is a dunite peridotite derived from mantle xenoliths in the San Carlos volcanic field, Arizona, USA. The host volcanism belongs to a 0.58 ± 0.21 Ma Miocene–Quaternary continental intraplate volcanic field in the Basin and Range–Colorado Plateau transition region[24]. San Carlos olivine in these xenoliths is widely recognized as Fo-rich. The xenolith suite also includes lherzolite and harzburgite[25], but those were not available in this study. The olivine grains do not show any alteration.
A05-Ol	Peridotite	Dunite peridotite, Jiaohe peridotite, Jihin Province	The sample is dunite derived from Jiaohe peridotite xenoliths hosted in Cenozoic alkali basalts from Jiaohe City, Jilin Province, NE China. Jiaohe lies near the northern segment of the Tan-Lu fault zone and represents continental intraplate alkaline volcanism linked to lithospheric extension and upper-mantle modification in NE China[26]. The xenolith suite is dominated by spinel lherzolite and harzburgite, but also includes dunite. The olivine is mantle-derived, with Fo contents

			of about 89–91 reported for the Jiaohe peridotites[26]. This sample is derived from subcontinental lithospheric mantle material entrained by the ascending host magma.
A06-Ol	Peridotite	Dunite peridotite, Kohistan Arc Pakistan	The sample is dunte derived from peridotitic ultramafic rocks of the Kohistan arc, NW Pakistan. The Kohistan arc is a Cretaceous intra-oceanic island arc formed during Neo-Tethyan subduction, with arc development broadly spanning ca. 134–90 Ma[27]. Peridotitic sections of the arc are represented by refractory harzburgite–dunite bodies. This sample is collected from dunitic peridotite. The olivine grains do not s how any alteration.
E02-Peri	Peridotite	Dunite, Gusdal olivine pit, Norway	The sample is dunite from the Gusdal Olivine Pit (Åheim). This pit is located within the Almklovdalen peridotite massif of the Western Gneiss Region, western Norway[28,29]. The massif represents mantle-derived ultramafic rock of Precambrian origin that was later exhumed and strongly overprinted during the Caledonian orogeny[29,30]. The sampled rock contains some phlogopite, but olivine is by far the dominant mineral. Under thin section, it does not show any alteration.
A12A-Peri	Peridotite	Dunite, Seqi Mine, Greenland	These three samples are from a dunitic peridotite from the Seqi Ultramafic Complex, Akia terrane, SW Greenland. The complex is hosted by tonalitic orthogneiss of the Mesoarchaean Akia terrane within the North Atlantic Craton and predates granitoid sheets dated at 2978 ± 8 Ma[31]. Tectonically, the Akia terrane forms part of an Archean crustal domain whose associated supracrustal belts are generally interpreted as arc-related / suprasubduction-zone assemblages. The Seqi body represents an Archean ultramafic cumulate body and consists of olivine-dominated plutonic ultramafic rocks, including dunite, other peridotite, and chromitite, and is interpreted as an ultramafic cumulate complex rather than a mantle fragment. These samples are collected from dunitic peridotite.
A12B-Peri	Peridotite	Dunite, Seqi Mine, Greenland	
A12C-Peri	Peridotite	Dunite, Seqi Mine, Greenland	
A14-Peri	Peridotite	Harzburgite, Balmuccia, Italy	The sample is harzburgite from the Balmuccia peridotite massif, Ivrea–Verbano Zone, Western Italian Alps. The Balmuccia peridotites are relatively fresh ultramafic rocks, dominated by spinel lherzolite with subordinate harzburgite and dunite, and are interpreted as fragments of subcontinental lithospheric mantle exposed within the lower-crustal Ivrea–Verbano Zone[32,33]. Their present exposure reflects Alpine exhumation of deep crust–mantle sections along major shear zones, particularly the Insubric Line[33]. Recent work suggests that accretion and melting of the Ivrea–Verbano mantle section may have been linked to back-arc rifting and mantle upwelling in the late Devonian[34]. The studied sample was collected from an abandoned quarry near the centre of the mapped Balmuccia peridotite body and consists mainly of olivine, orthopyroxene, and spinel, with minor clinopyroxene.
A03-Ol	Olivine separates	Basalt-hosted olivine, Hawaii	The sample is olivine separated from basaltic beach sand collected at South Point (Ka Lae), Hawaiʻi Island. South Point lies on the southern flank of Mauna Loa within the Hawaiian hotspot province, an ocean-island intraplate volcanic setting[35]. The olivine-rich sand is derived from nearby Quaternary basaltic volcanic deposits in the South Point–Puʻu Mahana area, with the principal local olivine-rich source commonly taken to be around 49–50 ka in age[36].
A13-Ol	Olivine separates	Lherzolite-hosted, Kilbourne Hole	The sample is an olivine separate derived from spinel lherzolite xenoliths from Kilbourne Hole, Potrillo volcanic field, New Mexico, USA. Kilbourne Hole is a Late Pleistocene volcanic maar, at about 80 ka, located within the Rio Grande rift and

			representing a continental intraplate extensional tectonic setting[3]. The mantle xenolith suite at Kilbourne Hole is dominated by spinel lherzolite, with subordinate harzburgite and dunite[37]. The olivine separate therefore represents older subcontinental lithospheric mantle material derived specifically from lherzolite xenoliths entrained by the host magma.
A16-Ol	Fayalite	Lastigkulla, Sweden	The sample is fayalitic olivine from the Lustigkulla Mine, Svartberg orefield, Väster Silvberg, Bergslagen district, Dalarna County, Sweden. Bergslagen is a Paleoproterozoic metavolcanic–metasedimentary ore province of the Fennoscandian Shield, with supracrustal rocks broadly dated at ca. 1.91–1.75 Ga and associated intrusive rocks at ca. 1.90–1.87 Ga[38]. Tectonically, Bergslagen is commonly interpreted as a volcanic-extensional to back-arc-related Paleoproterozoic province[39]. Minor additional unidentified minerals may also be present along with fayalitic olivine.
A04-Ol	Olivine separates	Admire pallasite meteorite, Arizona	The sample is an olivine separate from the Admire pallasite, a main-group stony–iron meteorite from Admire, Lyon County, Kansas, USA (Meteoritical Bulletin Database). Admire was found in 1881 and is classified as a main-group pallasite, a meteorite type composed chiefly of olivine crystals enclosed in Fe–Ni metal (Meteoritical Bulletin Database. Pallasites are widely interpreted as material derived from deep, differentiated parent bodies, commonly linked to the core–mantle boundary region of planetesimals, although their exact origin remains debated[40]. The sample used in this study consists only of olivine separates recovered from this Admire pallasitic meteorite.

Notes: Supplementary Fig. S1 shows photos of selected samples. Supplementary Data provides the whole rock composition of these samples.

Supplementary References

1. Baidya, A. S., Gehringer, M. M., Savaniu, C., Heubeck, C. & Stüeken, E. E. Magmatic and thermally produced reactive phosphorus 3.2 billion years ago and its implications for early life. *Commun. Earth Environ.* **6**, 1–13 (2025).

2. Thomas, L. E., Hawkesworth, C. J., Van Calsteren, P., Turner, S. P. & Rogers, N. W. Melt generation beneath ocean islands: a U-Th-Ra isotope study from Lanzarote in the Canary Islands. *Geochim. Cosmochim. Acta* **63**, 4081–4099 (1999).

3. Thompson, R. N. *et al.* Source of the Quaternary Alkalic Basalts, Picrites and Basanites of the Potrillo Volcanic Field, New Mexico, USA: Lithosphere or Convecting Mantle? *J. Petrol.* **46**, 1603–1643 (2005).

4. Nisbet, E. G. *et al.* Uniquely fresh 2.7 Ga komatiites from the Belingwe greenstone belt, Zimbabwe. *Geology* **15**, 1147–1150 (1987).

5. Stracke, A., Willig, M., Genske, F., Béguelin, P. & Todd, E. Major and trace element concentrations and Sr, Nd, Hf, Pb isotope ratios of global mid ocean ridge and ocean island basalts. *GFZ Data Services* (2022) doi:https://doi.org/10.5880/digis.e.2024.008.

6. Perkins, D. & Anthony, E. Y. The evolution of spinel lherzolite xenoliths and the nature of the mantle at Kilbourne Hole, New Mexico. *Contrib. to Mineral. Petrol.* **162**, 1139–1157 (2011).

7. Gómez-Ulla, A., Sigmarsson, O., Huertas, M. J., Devidal, J.-L. & Ancochea, E. The historical basanite - alkali basalt - tholeiite suite at Lanzarote, Canary Islands:

Carbonated melts of heterogeneous mantle source? *Chem. Geol.* **494**, 56–68 (2018).

8. Carracedo, J. C. *et al.* Hotspot volcanism close to a passive continental margin: the Canary Islands. *Geol. Mag.* **135**, 591–604 (1998).

9. Borzi, A. M., Giuffrida, M., Zuccarello, F., Palano, M. & Viccaro, M. The Christmas 2018 Eruption at Mount Etna: Enlightening How the Volcano Factory Works Through a Multiparametric Inspection. *Geochemistry, Geophys. Geosystems* **21**, e2020GC009226 (2020).

10. Schellart, W. P. Mount Etna–Iblean volcanism caused by rollback-induced upper mantle upwelling around the Ionian slab edge: An alternative to the plume model. *Geology* **38**, 691–694 (2010).

11. Viccaro, M. & Zuccarello, F. Mantle ingredients for making the fingerprint of Etna alkaline magmas: implications for shallow partial melting within the complex geodynamic framework of Eastern Sicily. *J. Geodyn.* **109**, 10–23 (2017).

12. Giuffrida, M., Cardone, M., Zuccarello, F. & Viccaro, M. Etna 2011–2022: Discoveries from a decade of activity at the volcano. *Earth-Science Rev.* **245**, 104563 (2023).

13. Janse van Rensburg, D. J., Heubeck, C. E. & Reimann, S. Volcanoes in the estuaries: Insights into Earth's oldest (3.22 Ga) terrestrial microbial habitats, Moodies Group, Barberton Greenstone Belt. *Precambrian Res.* **365**, 106325 (2021).

14. Heubeck, C. *et al.* Timing of deposition and deformation of the Moodies Group (Barberton Greenstone Belt, South Africa): Very-high-resolution of Archaean surface processes. *Precambrian Res.* **231**, 236–262 (2013).

15. Prendergast, M. D. & Wingate, M. T. D. Zircon geochronology of late Archean komatiitic sills and their felsic country rocks, south-central Zimbabwe: A revised age for the Reliance komatiitic event and its implications. *Precambrian Res.* **229**, 105–124 (2013).

16. Hofmann, A., Li, X., Bradley, D. C. & Humbert, F. The Belingwe greenstone belt, Zimbabwe – some new age constraints and implications for greenstone belt evolution. *Int. Geol. Rev.* **66**, 2940–2959 (2024).

17. Jerram, M. *et al.* The δ53Cr isotope composition of komatiite flows and implications for the composition of the bulk silicate Earth. *Chem. Geol.* **551**, 119761 (2020).

18. Donaldson, C. H. Origin of some of the Rhum harrisite by segregation of intercumulus liquid. *Mineral. Mag.* **45**, 201–209 (1982).

19. TROLL, V. R., NICOLL, G. R., DONALDSON, C. H. & EMELEUS, H. C. Dating the onset of volcanism at the Rum Igneous Centre, NW Scotland. *J. Geol. Soc. London.* **165**, 651–659 (2008).

20. Emeleus, C. H. & Troll, V. R. The Rum Igneous Centre, Scotland. *Mineral. Mag.* **78**, 805–839 (2014).

21. Larsen, J. G. Mantle-derived dunite and lherzolite nodules from Ubekendt Ejland, west Greenland Tertiary province. *Mineral. Mag.* **46**, 329–336 (1982).

22. Bernstein, S. & Brooks, C. K. Mantle xenoliths from Tertiary lavas and dykes on Ubekendt Ejland, West Greenland. *Geol. Greenl. Surv. Bull.* **180**, 152–154 (1998).

23. LARSEN, L. M. *et al.* Age of Tertiary volcanic rocks on the West Greenland continental margin: volcanic evolution and event correlation to other parts of the North Atlantic Igneous Province. *Geol. Mag.* **153**, 487–511 (2016).

24. Tilhac, R., Morishita, T., Hanaue, N., Tamura, A. & Guotana, J. M. Systematic LREE

enrichment of mantle harzburgites: The petrogenesis of San Carlos xenoliths revisited. *Lithos* **396–397**, 106195 (2021).

25. Lambart, S., Hamilton, S. & Lang, O. I. Compositional variability of San Carlos olivine. *Chem. Geol.* **605**, 120968 (2022).

26. Gan, W., Jin, Z., Fang, Z. & Liu, W. Deformation of the Subcontinental Lithospheric Mantle in NE China: Constraints from Rheological and Fabric Study of Mantle Peridotite Xenoliths from Jiaohe, Jilin Province. *J. Earth Sci.* **34**, 767–775 (2023).

27. Petterson, M. G. A Review of the geology and tectonics of the Kohistan island arc, north Pakistan. *The Evolving Continents: Understanding Processes of Continental Growth* vol. 338 0 (2010).

28. Cordellier, F., Boudier, F. & Boullier, A. M. Structural study of the Almklovdalen peridotite massif (southern Norway). *Tectonophysics* **77**, 257–281 (1981).

29. Medaris, L. G. A geothermobarometric investigation of garnet peridotites in the Western Gneiss Region of Norway. *Contrib. to Mineral. Petrol.* **87**, 72–86 (1984).

30. BEYER, E. E., GRIFFIN, W. L. & O'REILLY, S. Y. Transformation of Archaean Lithospheric Mantle by Refertilization: Evidence from Exposed Peridotites in the Western Gneiss Region, Norway. *J. Petrol.* **47**, 1611–1636 (2006).

31. Szilas, K. *et al.* Highly refractory Archaean peridotite cumulates: Petrology and geochemistry of the Seqi Ultramafic Complex, SW Greenland. *Geosci. Front.* **9**, 689–714 (2018).

32. Voshage, H. *et al.* Isotopic evidence from the Ivrea Zone for a hybrid lower crust formed by magmatic underplating. *Nature* **347**, 731–736 (1990).

33. Decarlis, A., Zanetti, A., Ogunyele, A. C., Ceriani, A. & Tribuzio, R. The Ivrea-Verbano tectonic evolution: The role of the crust-mantle interactions in rifting localization. *Earth-Science Rev.* **238**, 104318 (2023).

34. Ogunyele, A. C., Sanfilippo, A., Salters, V. J. M., Bonazzi, M. & Zanetti, A. Accretion of "young" Phanerozoic subcontinental lithospheric mantle triggered by back-arc extension—the case of the Ivrea-Verbano Zone. *Sci. Rep.* **14**, 11805 (2024).

35. Clague, D. A. & Dalrymple, G. B. The Hawaiian-Emperor volcanic chain. Part I. Geologic evolution. *US Geol. Surv. Prof. Pap.* **1350**, 5–54 (1987).

36. Trusdell, F. A. & Lockwood, J. *Geologic map of the southern flank of Mauna Loa Volcano, Island of Hawai'i, Hawaii*. *Scientific Investigations Map* (2020) doi:10.3133/sim2932C.

37. Satsukawa, T. *et al.* Seismic anisotropy of the uppermost mantle beneath the Rio Grande rift: Evidence from Kilbourne Hole peridotite xenoliths, New Mexico. *Earth Planet. Sci. Lett.* **311**, 172–181 (2011).

38. Frank, K. S. *et al.* Magnetite as a provenance and exploration tool for metamorphosed base-metal sulfide deposits in the Stollberg ore field, Bergslagen, Sweden. *Mineral. Mag. a J. Miner. Sci.* **86**, 373–396 (2022).

39. Ogenhall, E. Plate tectonic settings of the Svecofennian Palaeoproterozoic volcanic rocks at Hamrånge and Loos, south central Sweden, based on geochemical data. *GFF* **129**, 211–226 (2007).

40. Windmill, R. J. *et al.* Isotopic evidence for pallasite formation by impact mixing of olivine and metal during the first 10 million years of the Solar System. *PNAS Nexus* **1**, pgac015 (2022).
