# Supplementary material for: Mafic-ultramafic igneous rocks as a source of reactive phosphorus for the origin of life
Source: Nat Commun. 2026 Jul 23;17:8994. doi: 10.1038/s41467-026-75933-6 (PMC13507249; doi:10.1038/s41467-026-75933-6)
Supplement: Supplementary file 2 [file 41467_2026_75933_MOESM2_ESM.pdf]

Description of Additional Supplementary Files:

Supplementary Data 1: Whole rock data.

Supplementary data 2: Python code
