# Supplementary material for: Mafic-ultramafic igneous rocks as a source of reactive phosphorus for the origin of life
Source: Nat Commun. 2026 Jul 23;17:8994. doi: 10.1038/s41467-026-75933-6 (PMC13507249; doi:10.1038/s41467-026-75933-6)
Supplement: Supplementary file 3 [file 41467_2026_75933_MOESM5_ESM.pdf]

nature portfolio

Peer Review File

Mafic-ultramafic igneous rocks as a source of reactive phosphorus for the origin of life

Corresponding Author: Dr Abu Baidya

This file contains all reviewer reports in order by version, followed by all author rebuttals in order by version.

Version 0:

Reviewer comments:

Reviewer #1

(Remarks to the Author)
Due to the scarcity of phosphate, reduced phosphorus species with higher solubility and greater reactivity likely played an increasingly important role in the origin and evolution of life on early Earth. In contrast to recently emphasized sporadic sources (e.g., extraterrestrial delivery, lightning) or high-temperature redox processing, this study identifies a more fundamental and widespread reservoir. Through direct measurements, the authors demonstrate that the early Earth's abundant mafic-ultramafic rocks contain a significant amount of reduced P content. Using a box model with estimated flux from seafloor weathering and sinks from previous research, they further estimate the concentration of dissolved phosphite, suggesting that these mafic rocks are a critical primary P source on early Earth.
First, I acknowledge the significance and originality of this work. Although the presence of phosphite in mafic-ultramafic rocks has been hypothesized, a systematic, quantitative survey across diverse rock types and ages has been absent. The authors address this gap by employing a robust phosphorus extraction and speciation protocol, thereby revealing a substantial and previously underappreciated reservoir of reduced phosphorus. The subsequent box model effectively underscores the potential importance of this flux for phosphorus-starved early oceans. Thus, the quantitative characterization of phosphorus content and speciation in these rocks represents a valuable and necessary advance.
However, from a petrological and magmatic geochemistry perspective, several aspects of the study could be strengthened. The origin of the reduced phosphorus is not clearly explicated, and the interpretation of the differential P content between komatiites and basalts appears oversimplified. I suggest that mantle source characteristics, including its redox state, mineralogy, and the potential presence of accessory phosphides, are likely first-order controls. Developing a more comprehensive model that integrates these deep mantle processes with subsequent shallow crustal weathering would significantly improve the narrative. Such a model, spanning from source to sink, would not only refine the present analysis but also attract wider interest from researchers across deep-Earth geochemistry and surface biogeochemical cycling.
Additionally, certain descriptions in the rock geochemistry section could benefit from greater precision. Consulting with a specialist in igneous petrology or high-temperature geochemistry might help refine the terminology and strengthen the petrogenetic framework. Overall, this study provides compelling empirical evidence for an important planetary phosphorus source, and addressing these suggestions would further solidify its foundational contribution to the field.

General comments
1. When you say a suite of mafic to ultramafic rocks, we assume that these rocks are spatially and temporally linked and originate from a common tectono-magmatic event. These samples you chose worldwide cannot be strictly defined as a single "suite". It is recommended to use a more accurate term such as "a global compilation" or "a dataset" to describe your sample collection. Meanwhile, as these samples are widespread, please consider adding a comprehensive sample summary table. This table should include, at a minimum, the following fields for each: age, tectonic setting, and a brief note on petrogenesis. This would greatly facilitate reader comprehension by providing the necessary context for interpreting the geochemical data.
2. The authors observe that komatiites contain more P^{3+} (in the form of phosphite) than basalts and associate this with high temperature or higher compatibility of P^{3+}.
In a homogeneous, high-temperature silicate melt at ~1600°C, any redox equilibrium between P^{5+} and P^{3+} would be rapidly established, making it difficult to explain the preservation of a significant disequilibrium enrichment of P^{3+} solely by an in-

melt reduction process. The specific low-temperature solid-state reaction mechanism (cited by the author) is unlikely to be similarly operational in a fully molten or near-liquidus high-T environment.
Explanation 2 also requires further evaluation. The proposal of different bulk partition coefficients for P^{3+} and P^{5+} is testable. Would it be possible to plot the P^{3+} data (e.g., $P^{3+}/\Sigma P$ ratio) against the proxies for partial melting degree used in the study? Comparing the trends of P^{3+} and P^{5+} (or a reference incompatible element like La) on such a diagram would provide a much clearer and direct visual assessment of whether P^{3+} is indeed retained in the source more effectively during the melting process that produces komatiites vs. basalts.
Instead, I may suggest considering the mantle source composition. A more compelling model involves the incorporation of reduced phosphides from deep mantle reservoirs into the magma, followed by rapid quenching and incomplete oxidation during eruption to form phosphite (P^{3+}). Komatiitic melts, formed by higher degree melting of the deep and reduced mantle source, could efficiently entrain these phosphide grains. Upon eruption and rapid quenching, these phosphides undergo incomplete oxidation by water to form phosphite (P^{3+}), which may be preserved in glass or melt inclusions.

Line-by-line comments
L75–77: “These lithologies …”
The statement that magmatic mafic and ultramafic rocks are characterized by "high temperatures, low water activity, and high Fe^{2+} conditions" presents these features as a definitive and universal association. This is not accurate. Water activity can be highly variable, and the Fe^{2+}/Fe^{3+} ratio is a function of oxygen fugacity, which is not exclusively tied to rock type. While high temperatures are typical, they are not a defining “characteristic” in the same sense as composition. This description should be revised to reflect the potential range of conditions under which such rocks form.
L81–83: “Peridotite, …”
A little bit confusing description. A more precise statement would be: “Peridotite, particularly harzburgite and dunite, were included to probe the mantle reservoir, as they represent refractory residues formed after mantle partial melting. The melt composition (e.g., basalt or komatiite) is primarily governed by the extent and pressure-temperature conditions of melting.”
L98: “Earth’s oceanic crust”
Line 106: “the lowest” should be “lowest”
Line 107–108:
The observed discrepancy between whole-rock P content and P concentration in olivine separates suggests that olivine may not be the sole significant host of phosphorus in these ultramafic rocks. This discrepancy itself is a key finding, as it strongly indicates the presence of other significant P-bearing phases (like pyroxene, garnet, or glass?). To resolve this and move beyond inference, why not analyze both whole-rock and mineral separates (olivine and potentially orthopyroxene/clinopyroxene) from the same hand samples? This would allow for a mass-balance assessment, definitively identifying the major P reservoirs and strengthening the conclusions about P behavior.
L111–113:
The authors use a binary plot of total P versus K_2O (for all samples) to infer processes of “partial melting and magmatic differentiation.” This approach is conceptually flawed for a dataset of genetically unrelated rocks, as different source composition is the primary control on the absolute abundances of incompatible elements like P and To discuss melting or differentiation trends meaningfully, one must first isolate suites of rocks known to be co-genetic. For the present global dataset, I recommend reframing the conclusion to avoid implying a direct process link.
L136: Among the many incompatible elements, please clarify the specific rationale for selecting Mo as a comparator. Is it due to its particular sensitivity to redox conditions? A brief justification would be helpful. In addition, please plot P3+ data as well to show its correlation with incompatible elements and compare with PP5+.
L153–155:
As previously emphasized, these rocks do not constitute a cogenetic suite. Magmatic differentiation is a process that operates within a single, evolving magma batch or series.
L157–159:
A more convincing argument could be made by showing that U contents or U/Th ratios have no correlation with standard proxies for alteration (e.g., LOI).
L186–187: This should be Figure not Figure 3.
L188–194:
The authors suggest that weathering releases reduced P species from basaltic rocks and propose that potential sinks may include photochemical oxidation and dark oxidation of P^{3+}. Our recent work has revealed that phyllosilicate minerals formed during seafloor weathering processes can adsorb released phosphate, serving as an important sink in early Earth’s ocean. So have adsorption processes been considered? The interaction between seafloor weathering products and reduced phosphorus may require further exploration or clarification.
L210: For the lake system, riverine input of P3+ is the major source. But where does this riverine P3+ derive? You may need to state it more clearly.
L176 and L323:
When considering the temporal evolution of crustal reactive P, several key factors influencing reactive P availability appear underexplored: Firstly, the magmatic origin of reduced P, particularly its sensitivity to mantle oxygen fugacity, which evolved significantly from the early to late Archean. Secondly, the role of continental emergence and atmospheric oxygenation in shaping chemical weathering regimes and P speciation. Thirdly, the contribution of felsic continental crust, which is not addressed but may significantly influence the global P cycle. The proposed process seems more relevant to deep-sea alteration rather than surficial weathering, which should be clarified.
Table 1: “SD” should be explained in the table, and details about the deviation should be illustrated.
Figure 1: 1.5IQR in the legend should be explained in the figure caption.
Supplementary material: Please provide the whole dataset of the rock major and trace element compositions.

Xing Cui

The University of Hong Kong

(Remarks on code availability)

Reviewer #2

(Remarks to the Author)
In “Mafic-ultramafic igneous rocks as a source of reactive phosphorus for the origin of life” the authors demonstrate that the reduced oxidation state phosphorus oxyanion, phosphite, and the condensed P ion, pyrophosphate, are both ubiquitous and occasionally significant fractions of the P inventory of the more primitive igneous rocks. Broadly this finding is of significance to geochemistry, which generally presumes all P is in phosphate in terrestrial rocks, and as such it is generally significant and introduces a new perspective on phosphorus chemistry in igneous rocks.

This paper presents exciting new findings of phosphite in several rock types, with phosphite comprising up to 20+% of the total P in peridotites and komatiites. Pyrophosphate is lower in abundance in most samples. This is followed by a model discussing how this would accumulate on an early earth, reaching micromolar concentrations.

The paper is relatively straightforward and should be published with some further explanation and discussion.

Major comments:
1) One of the largest questions is why is the phosphite relatively consistent (in ppm) across rock types? Do the authors have control samples with no phosphite they can add to the paper to show this is not background contamination?

2) The paper speculates to some extent on the mechanism of phosphite formation/presence in these mafic materials, suggesting reduction of phosphate at high temperatures or that phosphite is more compatible in the mantle material. The paper could be strengthened by suggesting how determining the origin of phosphite might be better tested. For example, phosphite is present at room temperature as HXPO3X-3 where x >= 1. Do the authors expect that the phosphite extracted from the rocks is also present as the HXPO3X-3 ion in situ? Could it be present as phosphite (PO33-)? Lowe et al. (2025) demonstrated the production of this ion from trimetaphosphate and a strong reductant, and Chiu (2022) showed something similar using polyphosphates, mechanical grinding, and reductants. If the phosphite is present in the ultramafic/mafic materials as PO33-, then if extracted with D-labeled solvents, they might expect DXPO3X-3 but not if it is present as HXPO3X-3.

I would not expect the authors to perform these experiments as the ICPMS instrumental facilities could not make this determination, but if they provide some further information on how to tell apart different ions present in the rock, and what that might mean for the origin of the phosphite, that would be helpful. Additionally, discussion of the reduction of phosphate to phosphite from a condensed phosphate intermediate may illustrate that reduction is feasible.

The paper’s discussion of the origin of pyrophosphate is sufficient. I agree that the formation of pyrophosphate from incompatible phosphate (e.g., as part of glass) is reasonable.

3) The paper omitted reference to phosphite in serpentinites rocks (Pasek et al. 2022), which may be an additional source of phosphite on the early earth (as discussed in paragraph starting at line 55). Perhaps this serpentinite phosphite may be related to the findings in the present paper? Could the intrinsic, reduced phosphorus have been present in the precursor rocks to the serpentinites?

4) The model employs data from Ritson et al. (2020) for the UV oxidation of phosphite. Notably, the short oxidation timescale (<1 day) is in the presence of H2S/HS-. In the absence of sulfide, oxidation timescales are longer. This should be noted even if it doesn’t significantly change the findings of the paper.

Additionally, the paper uses the oxidative half-life for Fe(H2PO3)2 for the value of ‘dark oxidation’. This was established experimentally using a ferrous phosphite salt that contained water. The oxidation rate of phosphite in water is not well constrained, though Herschy et al. (2018) present a half-life of 103 years in water in air in the absence of UV. In anoxic conditions this may be a lot longer. The authors may want to consider a case where “Pdark_ox” is set to zero, or some other small number, given this large unknown.

Minor comment:
I might recommend changing the symbol for Pyrophosphate to PPi, as this is commonly used in biochemistry literature. Since there does not seem to be pyrophosphite (denoted as PPi(III)), this should be fine.

(Remarks on code availability)
I looked at the code but did not attempt to run it. My expectation is that the code will solve the problem stated but is not broadly applicable to other systems.

Reviewer #3

(Remarks to the Author)
This paper presents an analysis of phosphorus compounds within mafic and ultramafic rock samples from 15 locations around the world. Using the analytic results from their IC-ICP-MS experimental measurements and a box model based on a seafloor weathering flux model first presented by Sylverson et al, the authors make an argument for the availability of reactive phosphorus on the early Earth and other planetary bodies of interest to astrobiologists.

The method of extracting P-bearing compounds from rock samples using EDTA-NaOH has been widely used in previous studies, as cited in the paper. The IC-ICP-MS method is a relatively new (ca 2023) method developed and tested by several of the co-authors with supporting citations. IC-ICP-MS seems to be a robust method for detecting low limits of phosphorus-species.

The concentrations of P(III) shown in Table 1 for the various types of rocks are high enough (> 0.6 ppm according to Ref 25) that a more well-established corroborating method (e.g., P-NMR, uv-vis) could have been used to confirm those abundances. This would have strengthened the paper. In lieu of these measurements, a brief explanation of why they are not needed in this case would be helpful.

While the key conclusions of the paper rely on some assumptions, particularly with respect to the box model parameters, those assumptions are clearly stated and supported by citations to literature. Overall, the data appears to be robust, the analysis reproducible, and the interpretation reasonable.

Importantly, this paper establishes that reactive forms of phosphorus, especially reduced and polymerized phosphorus species such as phosphite and pyrophosphate, respectively, may be found in mafic and ultra-mafic rock samples that likely occur in meteorites and in the crusts of other planetary bodies. These more reactive forms of phosphorus promote the formation of phosphorus-bearing organic compounds, which would have been key ingredients for the emergence of life.

Moreover, this article establishes that these mafic and ultra-mafic rocks can release significant levels of reduced and polymerized phosphorus species due to weathering of seafloors, when photochemical oxidation is limited (i.e., up to 0.17 μM). The paper provides examples of key extraterrestrial environments where this water-rock interaction could occur and allow higher concentrations of reactive phosphorus to accumulate, including surface rocks on Mars in the past or on the rocky cores of Enceladus and Europa, where ice-layers could shield ultraviolet radiation.

Thus, this paper makes a strong case that mafic and ultra-mafic rocks in the seafloors and hydrothermal vents within prebiotic oceans could have been important reservoirs for reactive phosphorus contributing to the origin of life on Earth.

I recommend publishing the paper with the one minor suggestion made at the end of the third paragraph of this review.

(Remarks on code availability)
I believe that anyone with an understanding of Python should be able to use this code effectively. I was able to run the code in Google Colab to replicate Fig 3A in a few minutes.

Version 1:

Reviewer comments:

Reviewer #1

(Remarks to the Author)
Abu Saeed Baidya et al. have adequately addressed the major concerns in my previous comments. In particular, the statement regarding igneous rocks has been revised in a more appropriate manner, and the explanations concerning the relative proportions of phosphite have been clarified. Overall, this study makes an important contribution to our understanding of phosphorus bioavailability, especially in the context of early Earth environments. I therefore recommend publication after the authors address the following minor issues. Also, I do not need to review the manuscript again and am comfortable leaving the final decision to the editor.

Lines 130–132:
The sentence appears to be incomplete or unclear. Please revise this statement to improve its clarity and readability.

Line 136:
The rationale for selecting Mo as the representative incompatible trace element is not sufficiently explained. Since several incompatible trace elements could potentially be considered, please clarify why Mo was specifically chosen in this context.

Line 220–222:
The authors have appropriately removed the term “magmatic differentiation,” which addresses my main concern. However, the CaO–Al_2O_3 correlation may reflect primary lithological or mineralogical variation, but it should not be used as strong evidence for a magmatic trend or as definitive evidence against alteration. Please revise this statement in a more cautious and descriptive manner, like replacing “indicative of a magmatic trend” with “the positive correlation between CaO and Al_2O_3 is consistent with preservation of primary igneous/mineralogical compositional variations, rather than being dominated by secondary alteration…”

(Remarks on code availability)

Reviewer #3

(Remarks to the Author)
The authors have sufficiently addressed the comments of all three reviewers, and I recommend publication of this revised manuscript.

(Remarks on code availability)
None noted.

Open Access This Peer Review File is licensed under a Creative Commons Attribution 4.0 International License, which permits use, sharing, adaptation, distribution and reproduction in any medium or format, as long as you give appropriate credit to the original author(s) and the source, provide a link to the Creative Commons license, and indicate if changes were made.
In cases where reviewers are anonymous, credit should be given to 'Anonymous Referee' and the source.
The images or other third party material in this Peer Review File are included in the article's Creative Commons license, unless indicated otherwise in a credit line to the material. If material is not included in the article's Creative Commons license and your intended use is not permitted by statutory regulation or exceeds the permitted use, you will need to obtain permission directly from the copyright holder.
To view a copy of this license, visit https://creativecommons.org/licenses/by/4.0/

We thank the reviewers and the handling editor for their thoughtful and constructive comments on our manuscript. We are encouraged that the reviewers recognized the novelty and significance of documenting reactive phosphorus species in mafic–ultramafic igneous rocks and of evaluating their possible role as a sustained geological source of reactive phosphorus through seafloor weathering on the early Earth.

In response to the reviews, we have revised the manuscript substantially and provide a detailed point-by-point response (blue text) below. In particular, we have strengthened the manuscript in four main ways. First, we have added IC–ICP–MS control data, including raw data for starting materials, controls, and selected samples, to address concerns that the detected phosphite signal might reflect analytical background or contamination rather than a true sample signal (Fig. 1, Supplementary Material Fig. S2). Second, we have improved the geological and petrological description of the sample set by revising terminology and adding clearer contextual information on sample age, tectonic setting, and petrological characteristics (See Supplementary Table 1). Third, we have expanded the discussion of phosphite occurrence in the studied rocks, with a more careful treatment of possible controls on its abundance and the limitations of the present dataset in resolving its origin (See elaborate response to Reviewer 1). Fourth, we have clarified the assumptions, scope, and uncertainty of the box model, particularly with respect to oxidation pathways and adsorption sinks and related simplifications. We believe that these revisions have improved the clarity, rigor, and overall balance of the manuscript, and we are grateful to the reviewers for helping us strengthen the study.

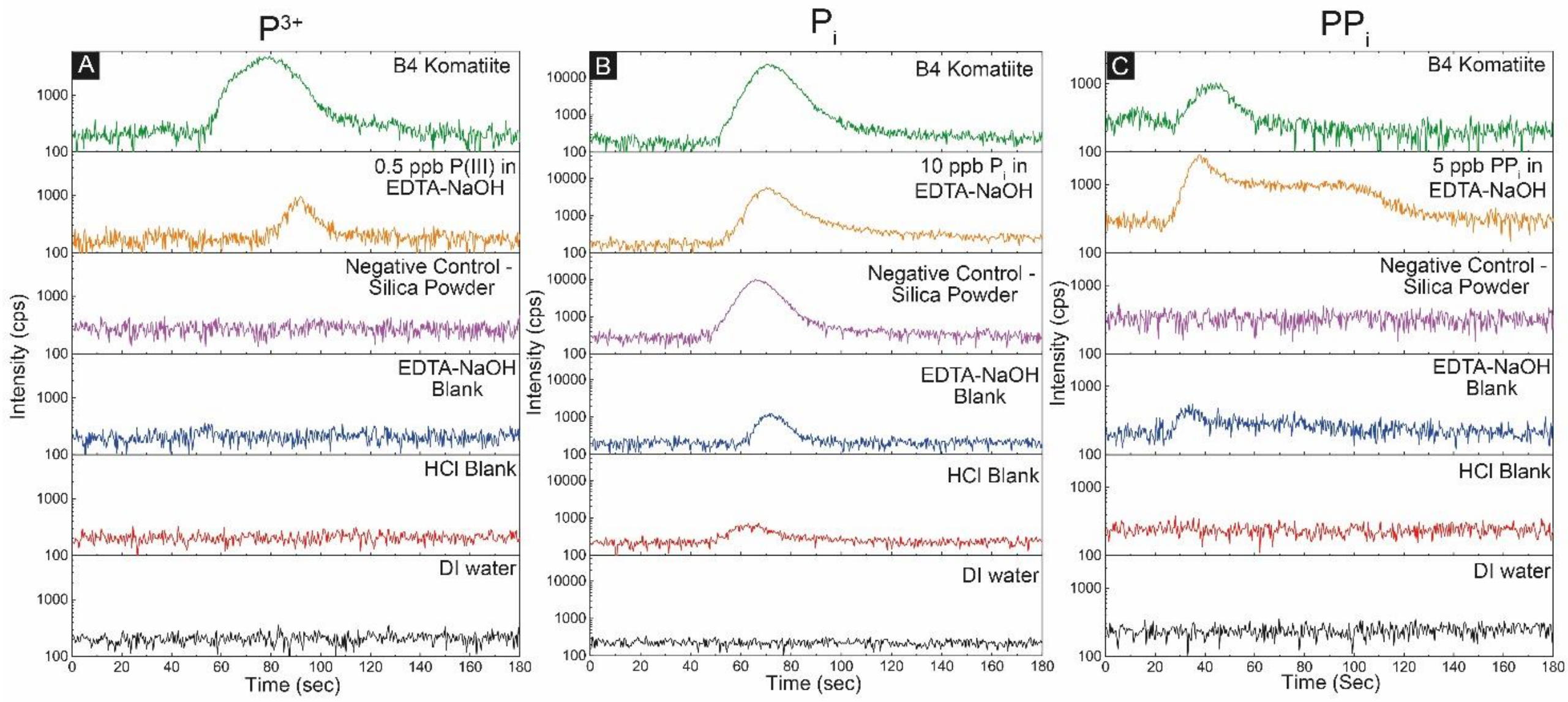

Figure 1: IC–ICP–MS chromatograms of P(III), Pi, and PPi in starting materials, blanks and selected samples. *A: No detectable P(III) is observed in DI water, washing HCl or the EDTA–NaOH extractant. Silica powder, prepared from powdered sea sand baked at 500 °C in air to oxidize any pre-existing P(III), also shows no detectable phosphite. By contrast, the 0.5 ppb P(III) standard and a representative komatiite sample show clear phosphite peaks. B: Phosphate (Pi) is detected in all starting materials except DI water; blank contributions from EDTA–NaOH were subtracted from sample values. C: No detectable pyrophosphate (PP_i) is observed in DI water or HCl, whereas EDTA–NaOH contains a minor PPi blank that was corrected for in the analyses of the samples.*

Reviewer #1 (Remarks to the Author):

Due to the scarcity of phosphate, reduced phosphorus species with higher solubility and greater reactivity likely played an increasingly important role in the origin and evolution of life on early Earth. In contrast to recently emphasized sporadic sources (e.g., extraterrestrial delivery, lightning) or high-temperature redox processing, this study identifies a more fundamental and widespread reservoir.

Through direct measurements, the authors demonstrate that the early Earth's abundant mafic-ultramafic rocks contain a significant amount of reduced P content. Using a box model with estimated flux from seafloor weathering and sinks from previous research, they further estimate the concentration of dissolved phosphite, suggesting that these mafic rocks are a critical primary P source on early Earth.

First, I acknowledge the significance and originality of this work. Although the presence of phosphite in mafic-ultramafic rocks has been hypothesized, a systematic, quantitative survey across diverse rock types and ages has been absent. The authors address this gap by employing a robust phosphorus extraction and speciation protocol, thereby revealing a substantial and previously underappreciated reservoir of reduced phosphorus. The subsequent box model effectively underscores the potential importance of this flux for phosphorus-starved early oceans. Thus, the quantitative characterization of phosphorus content and speciation in these rocks represents a valuable and necessary advance.

However, from a petrological and magmatic geochemistry perspective, several aspects of the study could be strengthened. The origin of the reduced phosphorus is not clearly explicated, and the interpretation of the differential P content between komatiites and basalts appears oversimplified. I suggest that mantle source characteristics, including its redox state, mineralogy, and the potential presence of accessory phosphides, are likely first-order controls. Developing a more comprehensive model that integrates these deep mantle processes with subsequent shallow crustal weathering would significantly improve the narrative. Such a model, spanning from source to sink, would not only refine the present analysis but also attract wider interest from researchers across deep-Earth geochemistry and surface biogeochemical cycling.

Response: We thank the reviewer for this thoughtful and constructive assessment and for recognizing the novelty and significance of the study. We agree that the original manuscript required a more careful petrological framing, particularly regarding the origin of reduced phosphorus and the interpretation of differences among lithologies. In the revised manuscript, we have accordingly expanded the discussion of possible controls, particularly related to mantle redox potential, mineralogy, and the presence of phosphide mineral, on phosphite occurrence in basalt and komatiites. Thus, the revised interpretation integrates deep mantles processes with subsequent shallow crustal weathering. These revisions are reflected in more detail in the following responses.

Additionally, certain descriptions in the rock geochemistry section could benefit from greater precision. Consulting with a specialist in igneous petrology or high-temperature geochemistry might help refine the terminology and strengthen the petrogenetic framework. Overall, this study provides compelling empirical evidence for an important planetary phosphorus source, and addressing these suggestions would further solidify its foundational contribution to the field.

Response: We agree that several descriptions in the rock geochemistry section required greater precision. In the revised manuscript, we have therefore revised the terminology and petrogenetic framing throughout to improve accuracy, clarify the geological context of the samples, and avoided language that could imply stronger genetic relationships or process-based interpretations than are supported by this globally distributed dataset. The changes are reflected in the Supplementary Table 1. We believe these revisions have improved the clarity and technical precision of the manuscript.

General comments

1. When you say a suite of mafic to ultramafic rocks, we assume that these rocks are spatially and temporally linked and originate from a common tectono-magmatic event. These samples you chose worldwide cannot be strictly defined as a single "suite". It is recommended to use a more accurate term such as "a global compilation" or "a dataset" to describe your sample collection. Meanwhile, as these samples are widespread, please consider adding a comprehensive sample summary table.

This table should include, at a minimum, the following fields for each: age, tectonic setting, and a brief note on petrogenesis. This would greatly facilitate reader comprehension by providing the necessary context for interpreting the geochemical data.

Response: We agree that the term “suite” was not appropriate for this globally distributed and genetically unrelated sample set. In the revised manuscript, we have replaced this terminology with more accurate wording such as “global compilation” or “dataset,” as recommended. We have also expanded the sample descriptions to provide clearer geological context, including available information on age, tectonic setting, and petrogenetic characteristics in the revised Supplementary Table 1. These revisions improve clarity and avoid implying a shared tectono-magmatic origin among the studied rocks.

2. The authors observe that komatiites contain more P^{3+} (in the form of phosphite) than basalts and associate this with high temperature or higher compatibility of P^{3+}. In a homogeneous, high-temperature silicate melt at ~1600°C, any redox equilibrium between P^{5+} and P^{3+} would be rapidly established, making it difficult to explain the preservation of a significant disequilibrium enrichment of P^{3+} solely by an in-melt reduction process. The specific low-temperature solid-state reaction mechanism (cited by the author) is unlikely to be similarly operational in a fully molten or near-liquidus high-T environment.

Explanation 2 also requires further evaluation. The proposal of different bulk partition coefficients for P^{3+} and P^{5+} is testable. Would it be possible to plot the P^{3+} data (e.g., $P^{3+}/\Sigma P$ ratio) against the proxies for partial melting degree used in the study? Comparing the trends of P^{3+} and P^{5+} (or a reference incompatible element like La) on such a diagram would provide a much clearer and direct visual assessment of whether P^{3+} is indeed retained in the source more effectively during the melting process that produces komatiites vs. basalts.

Instead, I may suggest considering the mantle source composition. A more compelling model involves the incorporation of reduced phosphides from deep mantle reservoirs into the magma, followed by rapid quenching and incomplete oxidation during eruption to form phosphite (P^{3+}). Komatiitic melts, formed by higher degree melting of the deep and reduced mantle source, could efficiently entrain these phosphide grains. Upon eruption and rapid quenching, these phosphides undergo incomplete oxidation by water to form phosphite (P^{3+}), which may be preserved in glass or melt inclusions.

Response: We thank the reviewer for this important suggestion. Our analyses show that P(III)/ΣP is much higher in komatiites, peridotites, and olivine separates than in basalts, whereas the absolute P(III) concentrations overlap more strongly because basalts contain substantially more total P overall. Specifically, average P(III)/ΣP is 0.22% in basalt, 4.89% in komatiite, 5.33% in olivine separates, and 5.52% in peridotite, while the corresponding average absolute P(III) concentrations are 4.78 ppm, 4.37 ppm, 1.46 ppm, and 0.97 ppm, respectively. We therefore now state explicitly that the key contrast in our dataset is the relative enrichment of reduced P, not simply its absolute abundance.

We attributed this difference to temperature and/or incompatible behaviour of P(III). Regarding temperature: Previous experiments at solid state (Baidya et al. 2024), as well as thermodynamic models (see below, Herschey et al. 2018) have shown that increasing temperature tends to favor a higher P(III)/P(V) ratio. No experiments have so far been conducted under magmatic temperatures of 1600 °C; however, if the trend holds, then melts approaching such a high temperature (such as komatiites) may also express a higher P(III)/P(V) ratio at equilibrium than lower-temperature melts (such as basalts). Importantly, the reduction of P(V) to P(III) is not a disequilibrium process. Hence it is conceivable that temperature plays a role in setting the P(III)/P(V) ratio of the rock (in concert with fO_2). We would therefore like to keep this point in the manuscript.

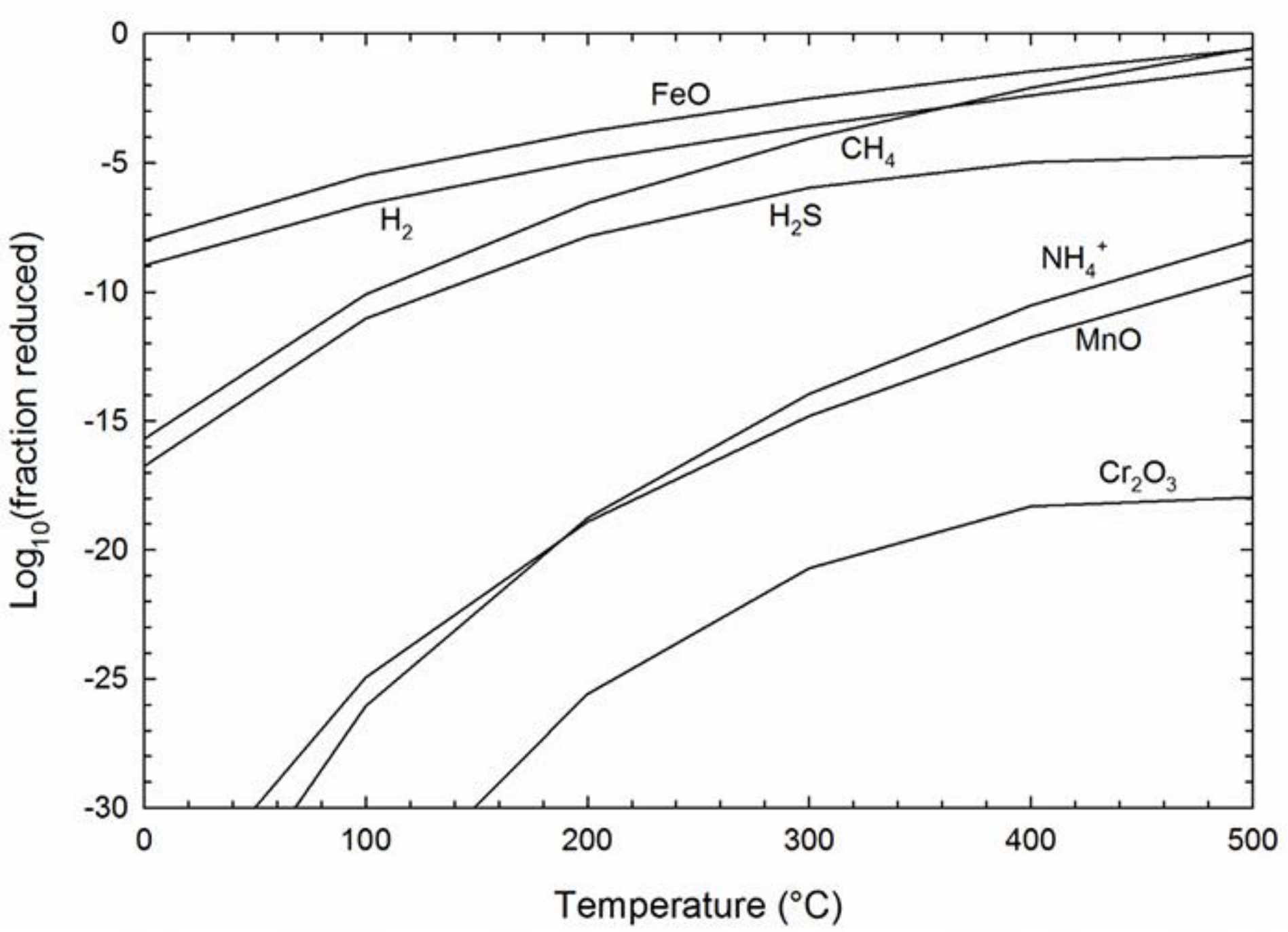

Figure 2: taken from Herschey et al. 2018 (Nature Communications). 'Fraction reduced' on the y-axis is equivalent to the P(III)/ΣP ratio. The plot shows that with increasing temperature and in equilibrium with a given reductant, such as FeO, the equilibrium P(III)/ΣP ratio increases.

Regarding compatibility: To explore whether differences in the bulk partitioning behaviour of P(III) and P_i may contribute to the observed pattern, we examined the relationship between P(III)/ΣP and differentiation-sensitive trace elements, particularly La and Ni. La was chosen because it behaves incompatibly and is enriched in basalts relative to komatiites, whereas Ni shows the opposite trend. The observed anticorrelation between P(III)/ΣP and La, together with the positive correlation with Ni (Fig. 2; Supplementary Fig. S6), is consistent with the possibility that P(III) behaves more compatibly than P_i during mantle melting and magmatic evolution. We acknowledge that there is significant scatter in these plots, likely because the sample do not represent genetically related suites, as noted earlier. However, the magmatic behaviours of La and Ni are well-known and therefore provide a means of arranging our samples at least approximately from least to most differentiated. Our data also suggest that olivine can host P(III). If so, the higher degrees of mantle melting required to generate komatiites, and the associated greater involvement of olivine-bearing mantle, may have promoted greater release of P(III) from the source, thereby contributing to the elevated P(III)/ΣP ratios in komatiites.

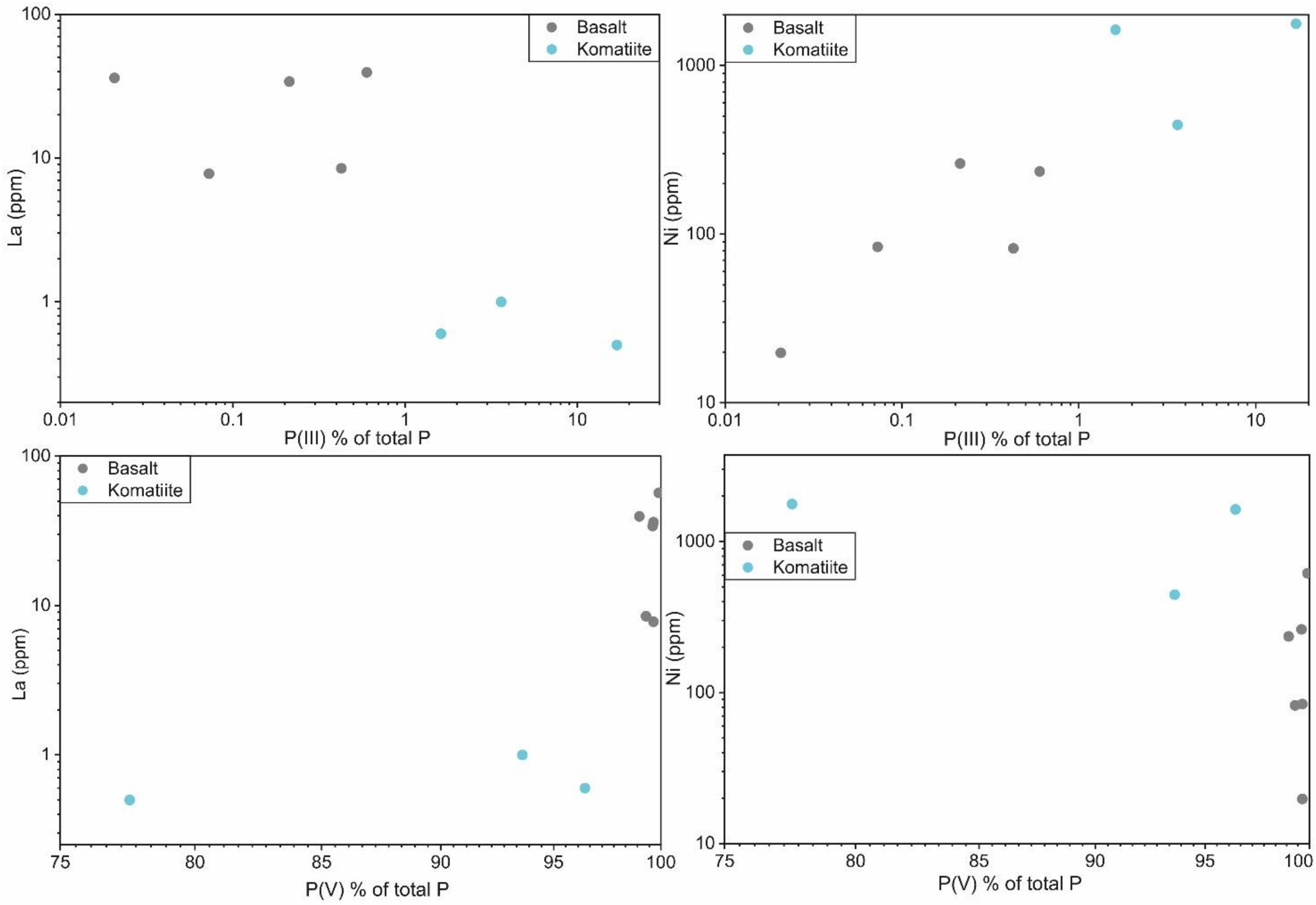

***Figure 3: Relationship between magmatic differentiation indicators (La and Ni) and P(III)% and P_i%.** P(III)% shows moderate negative correlation with La and moderate positive correlation with Ni, whereas P_i% does not show the same relationships. This pattern is consistent with the possibility that P(III) behaves more compatibly than P_i during magmatic differentiation.*

In addition to magmatic differentiation and compatibility, mantle characteristics may control this pattern, as suggested by the reviewer. First, source redox may contribute to P(III)/ΣP ratio of a part of the ultramafic dataset. Our peridotite and olivine separates come from multiple mantle sources and tectonic settings, so it is reasonable that some may sample or preserve relatively reduced mantle domains. This is consistent with the fact that upper-mantle oxygen fugacity is heterogeneous over several log units, from about FMQ−3 to FMQ+1, with some cratonic Archean lithosphere extending even lower[1,2]. However, for the komatiite samples, source redox is likely not a controlling factor, because the Belingwe komatiites used in this study have been interpreted as derived from a source oxidation state broadly similar to present-day MORB-source mantle, based on melt-inclusion $Fe^{3+}/\Sigma Fe$ ratios[3]. We therefore revised the manuscript to treat mantle redox as a contributing control on the higher P(III)/ΣP in some ultramafic samples, rather than as a single explanation for all samples.

Second, we now explain more clearly how olivine or other silicate host phases can raise P(III)/ΣP. In basalt, bulk P is high, but the basalt-hosted olivine separate is P-poor relative to bulk basalt, showing that most basaltic P is not stored in olivine. By contrast, peridotites and olivine separates are olivine-rich, low-P residues, so even a modest reduced-P component retained in silicate domains would make up a much larger fraction of the total P inventory. This provides a direct explanation for why P(III)/ΣP is high in peridotite, komatiites and olivine separates but low in basalt: in ultramafic, olivine-rich rocks the total P reservoir is small, so any preserved reduced-P pool becomes proportionally important, whereas in basalt the much larger oxidized P reservoir dilutes the phosphite fraction. This interpretation is consistent with experimental studies showing that phosphorus can partition into olivine, clinopyroxene, and silicate glass, not only into phosphate minerals[4], and that olivine–melt P partitioning can vary strongly with melt composition[5].

Third, following the reviewer's suggestion, we now discuss a phosphide-mediated source pathway, particularly for the komatiite samples. Specifically, we note that deeper and higher-degree melting may entrain small phosphide-bearing reduced-P domains from the mantle, which could contribute to the elevated phosphite fraction observed in komatiites. Phosphides have been reported from mantle-hosted podiform chromitites, showing that highly reduced P domains can occur in mantle-derived settings[6]. If present in the mantle source of Belingwe komatiites, such phosphides may undergo partial oxidation during eruption and quenching in the presence of H_2O and produce P(III).

Overall, the elevated P(III)/ΣP in peridotite and olivine separates is best explained by preservation of reduced P in olivine-rich, low-P ultramafic residues and temperature, whereas the higher P(III)/ΣP in komatiite relative to basalt likely reflects a combination of this silicate-host effect with deeper melting and, potentially, contribution from phosphide-bearing reduced domains. These scientific points are now incorporated into the revised Discussion (Lines 125-172).

Line-by-line comments

L75–77: "These lithologies …"
The statement that magmatic mafic and ultramafic rocks are characterized by "high temperatures, low water activity, and high Fe^{2+} conditions" presents these features as a definitive and universal association. This is not accurate. Water activity can be highly variable, and the Fe^{2+}/Fe^{3+} ratio is a function of oxygen fugacity, which is not exclusively tied to rock type. While high temperatures are typical, they are not a defining "characteristic" in the same sense as composition. This description should be revised to reflect the potential range of conditions under which such rocks form.

Response: We agree that the original wording was too general. In the revised manuscript, we have revised this sentence to avoid presenting these features as universal characteristics of all mafic–ultramafic magmas. The text now more carefully states that such rocks commonly form under high-temperature conditions and are characterized by variable but often low water activity and Fe^{2+}/Fe^{3+} ratios.

L81–83: "Peridotite, …" A little bit confusing description. A more precise statement would be: "Peridotite, particularly harzburgite and dunite, were included to probe the mantle reservoir, as they represent refractory residues formed after mantle partial melting. The melt composition (e.g., basalt or komatiite) is primarily governed by the extent and pressure-temperature conditions of melting."

Response: We agree that the original wording was imprecise. In the revised manuscript, we have clarified that the peridotite samples, particularly harzburgitic and dunitic lithologies, were included to probe the mantle reservoir because they represent refractory residues of mantle melting that has led to the formation of basaltic and komatiitic melt compositions.

L98: "Earth's oceanic crust"

Response: We have corrected the wording in the revised manuscript.

Line 106: "the lowest" should be "lowest"

Response: The text has been corrected in the revised manuscript.

Line 107–108: The observed discrepancy between whole-rock P content and P concentration in olivine separates suggests that olivine may not be the sole significant host of phosphorus in these ultramafic rocks. This discrepancy itself is a key finding, as it strongly indicates the presence of other significant P-bearing phases (like pyroxene, garnet, or glass?). To resolve this and move beyond inference, why not analyze both whole-rock and mineral separates (olivine and potentially

orthopyroxene/clinopyroxene) from the same hand samples? This would allow for a mass-balance assessment, definitively identifying the major P reservoirs and strengthening the conclusions about P behavior.

Response: We agree that the discrepancy between whole-rock and olivine-separate P contents suggests that olivine is unlikely to be the only significant host of phosphorus in these ultramafic samples. In the revised manuscript, we now state this point more explicitly and note that additional P-bearing phases may contribute to the bulk-rock inventory. We agree that paired analysis of whole rock and multiple mineral separates from the same hand specimen would provide a stronger mass-balance constraint; however, this is beyond the scope of the present study, because other minerals besides olivine were too fine-grained in the samples that we had available and could therefore not be separated mechanically. But we anticipate that our work will motivate more research in this direction.

L111–113: The authors use a binary plot of total P versus K_2O (for all samples) to infer processes of "partial melting and magmatic differentiation." This approach is conceptually flawed for a dataset of genetically unrelated rocks, as different source composition is the primary control on the absolute abundances of incompatible elements like P and K. To discuss melting or differentiation trends meaningfully, one must first isolate suites of rocks known to be co-genetic. For the present global dataset, I recommend reframing the conclusion to avoid implying a direct process link.

Response: The reviewer is correct in saying that plots such as P species versus K can provide the erroneous impression of a genetically related suite, which was not our intention. However, in an earlier comment the reviewer requested plots of P versus differentiation indicators, for which we chose La and Ni. Such plots can still be useful, because elements such as K, La, and Ni have well-established magmatic behaviours and can therefore be used to quantitatively illustrate the maficity or degree of differentiation of an igneous rock. We would therefore like to keep these plots in the paper; however, we have now made it clear in the figure caption and discussion that no genetic relationship exists, which likely explains the scatter in the data.

L136: Among the many incompatible elements, please clarify the specific rationale for selecting Mo as a comparator. Is it due to its particular sensitivity to redox conditions? A brief justification would be helpful. In addition, please plot P3+ data as well to show its correlation with incompatible elements and compare with PP5+.

Response: In the revised manuscript, we now clarify the rationale for using Mo and K as a comparator, namely that they are incompatible elements therefore show an enrichment in basalts compared to ultramafic rocks and minerals. These elements provide a useful point of comparison for discussing P behaviour, which also behaves incompatibly. We have also plotted P(III)/ΣP with other incompatible elements such as La, which show a negative correlation possibly indicating its compatible behaviour (see discussion above). We also have plotted PP_i concentration against Na, K, and La, and the relationship is positive consistent with our interpretation that higher Pi concentration may facilitate Pi-Pi interaction and therefore, PP_i formation. These plots and above discussion also suggest that P(III) and PP_i may behave differently during magmatic differentiation or may form at different stages of magmatic differentiation.

L153–155: As previously emphasized, these rocks do not constitute a cogenetic suite. Magmatic differentiation is a process that operates within a single, evolving magma batch or series.

Response: Please see our response to L111–113. We agree that the studied samples are not genetically related however they still show a good correlation between CaO and Al2O3, which would not be the case if secondary alteration. In the revised manuscript, we have made this clear and removed the word 'magmatic differentiation'.

L157–159: A more convincing argument could be made by showing that U contents or U/Th ratios have no correlation with standard proxies for alteration (e.g., LOI).

Response: Unfortunately, we do not have LOI data available for these samples. Our main argument here is that the studied samples do not show any alteration and P(III) and PP_i signature is magmatic. First. we do not see any evidence of alteration in the studied basalt, peridotite, and olivine samples in selected thin sections and fluid-mobile elements particularly K and U show positive relationship is maintained (Supplementary Fig. S1 and S5). The komatiite samples studied also show minor or no alteration, documented independently in other studies[7]. We therefore find no indication of fluid alteration-induced formation of phosphite in our samples.

L186–187: This should be Figure 4, not Figure 3.

Response: The figure citation has been corrected in the revised manuscript.

L188–194: The authors suggest that weathering releases reduced P species from basaltic rocks and propose that potential sinks may include photochemical oxidation and dark oxidation of P^{3+}. Our recent work has revealed that phyllosilicate minerals formed during seafloor weathering processes can adsorb released phosphate, serving as an important sink in early Earth's ocean. So, have adsorption processes been considered? The interaction between seafloor weathering products and reduced phosphorus may require further exploration or clarification.

Response: We appreciate the review's concern that adsorption onto seafloor-weathering products may represent an additional sink for dissolved P(III); however, we think that this sink is likely minor in comparison to oxidation. In our previous work, we found that P(III) adsorption onto ferrihydrite is limited[8], and for this reason ferrihydrite adsorption was not treated as a major sink in the present model. Adsorption of P(III) onto other secondary minerals, including clays and related alteration products, has not yet been constrained, but considering that P_i adsorption on clays is lower or similar[9] to P_i adsorption on ferrihydrite, we suspect that P(III) adsorption on clays is also minor, similar to P(III) adsorption on Fe-oxides. We have revised the manuscript to clarify that such adsorption processes are not explicitly included and remain a source of uncertainty in this first-order framework. If adsorption onto weathering products is significant, then the P(III) concentrations estimated by the model would represent upper-bound values. However, given the argument described above, we do not think that this would significantly impact our results.

L210: For the lake system, riverine input of P3+ is the major source. But where does this riverine P3+ derive? You may need to state it more clearly.

Response: We thank the reviewer for pointing this out. In the revised manuscript, we now clarify that the riverine P(III) input in the lake model is derived from catchment weathering of mafic–ultramafic source rocks containing reduced phosphorus, and we have revised the text accordingly.

L176 and L323: When considering the temporal evolution of crustal reactive P, several key factors influencing reactive P availability appear underexplored: Firstly, the magmatic origin of reduced P, particularly its sensitivity to mantle oxygen fugacity, which evolved significantly from the early to late Archean. Secondly, the role of continental emergence and atmospheric oxygenation in shaping chemical weathering regimes and P speciation. Thirdly, the contribution of felsic continental crust, which is not addressed but may significantly influence the global P cycle. The proposed process seems more relevant to deep-sea alteration rather than surficial weathering, which should be clarified.

Response: We thank the reviewer for this important comment. We agree that reactive phosphorus availability through time would ultimately have been influenced by additional factors beyond those considered here, including secular changes in mantle redox state, continental emergence, atmospheric oxygenation, and contributions from felsic crust. However, the focus of the present model is on prebiotic and early biological conditions, where P(III) was perhaps most relevant for biochemistry. It is therefore designed as a first-order framework for estimating reactive P associated with mafic–ultramafic oceanic lithologies and released during anoxic seafloor weathering, as it would

have existed on the early Earth. We have revised the manuscript to clarify this scope more explicitly and to distinguish this process from continental and surficial weathering regimes. We also now state more clearly that the temporal basalt–komatiite proportions used in the model are only a first-order proxy, as direct constraints on the composition of Archean oceanic crust remain limited. The potential influence of felsic crust and other larger-scale environmental changes is now acknowledged as an important uncertainty and avenue for future work; however, we emphasize that felsic crust is not directly relevant to the seafloor weathering and therefore not directly related to our main objective.

Table 1: “SD” should be explained in the table, and details about the deviation should be illustrated.

Response: In the revised manuscript/Table 1 caption, we now define SD explicitly as standard deviation and clarify how the reported variation was calculated.

Figure 1: 1.5IQR in the legend should be explained in the figure caption. Supplementary material: Please provide the whole dataset of the rock major and trace element compositions.

Response: In the revised Supplementary Information, we have provided the full available dataset of major and trace element compositions for the studied rocks.

Xing Cui
The University of Hong Kong

Reviewer #2 (Remarks to the Author):

In “Mafic-ultramafic igneous rocks as a source of reactive phosphorus for the origin of life” the authors demonstrate that the reduced oxidation state phosphorus oxyanion, phosphite, and the condensed P ion, pyrophosphate, are both ubiquitous and occasionally significant fractions of the P inventory of the more primitive igneous rocks. Broadly this finding is of significance to geochemistry, which generally presumes all P is in phosphate in terrestrial rocks, and as such it is generally significant and introduces a new perspective on phosphorus chemistry in igneous rocks.

This paper presents exciting new findings of phosphite in several rock types, with phosphite comprising up to 20+% of the total P in peridotites and komatiites. Pyrophosphate is lower in abundance in most samples. This is followed by a model discussing how this would accumulate on an early earth, reaching micromolar concentrations.

The paper is relatively straightforward and should be published with some further explanation and discussion.

Response: We thank the reviewer for this positive and constructive assessment of our manuscript. We appreciate the reviewer’s recognition that the detection of phosphite and pyrophosphate in mafic–ultramafic rocks introduces a new perspective on phosphorus geochemistry in igneous systems and may have important implications for prebiotic environments. We also appreciate the reviewer’s specific suggestions for strengthening the manuscript. In the revised version, we have addressed these comments by adding IC–ICP–MS control data to clarify that the phosphite signal is not due to analytical background or contamination, expanding the discussion of the possible chemical form and origin of phosphite in the studied rocks, clarifying the relationship between our results and previous work on serpentinites, and revising the box-model discussion to better explain the assumptions and uncertainties associated with oxidation sinks. These revisions are detailed in the responses below.

Major comments:
1) One of the largest questions is why is the phosphite relatively consistent (in ppm) across rock types? Do the authors have control samples with no phosphite they can add to the paper to show this is not background contamination?

Response: We agree that the relatively similar absolute phosphite concentrations across several lithologies require clarification, and that it is also essential to demonstrate that the observed signal is not due to analytical background or contamination. In the revised manuscript, we now clarify that the absolute bulk-rock concentrations of P(III) were not measured directly in the solids, but were estimated by combining the P(III)/P_{total} ratio determined in the extract with the total P concentration of the corresponding rock sample and the extraction yield, following the approach of previous researchers[10]. As a result, the basalt samples, although they contain lower P(III)/P_{total} ratios than komatiites and ultramafic rocks, can still yield comparable absolute P(III) concentrations because their total P contents are substantially higher. Our approach is overall justified, because we did not see any relation between extraction yields and P(III)/ΣP ratio, which indicates that there was no preferential extraction of P(III) and Pi (Fig. 3). We have revised the text to make this calculation and its consequence clearer. In addition, we have added IC–ICP–MS control data for reagents, salts, and selected samples to show that the observed phosphite signal is distinct from the analytical background and is therefore not attributable to contamination (Fig. 1). These changes are now described in Lines 188-190 and 193-196.

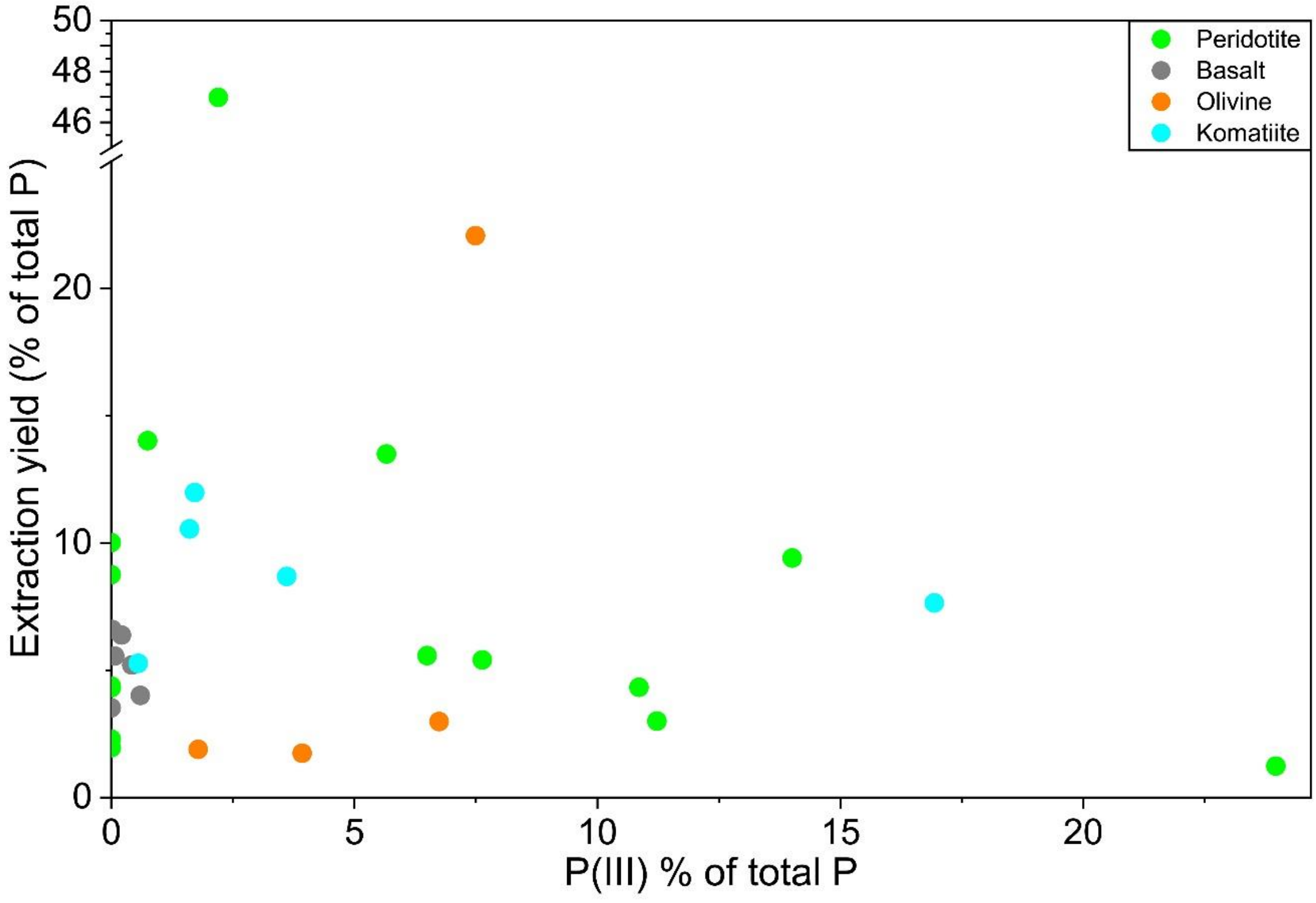

Figure 4: Binary plot shows no correlation between P(III)% and extraction yields.

2) The paper speculates to some extent on the mechanism of phosphite formation/presence in these mafic materials, suggesting reduction of phosphate at high temperatures or that phosphite is more compatible in the mantle material. The paper could be strengthened by suggesting how determining the origin of phosphite might be better tested. For example, phosphite is present at room temperature as HXPO3X-3 where x >= 1. Do the authors expect that the phosphite extracted from the rocks is also present as the HXPO3X-3 ion in situ? Could it be present as phosphite (PO33-)? Lowe et al. (2025) demonstrated the production of this ion from trimetaphosphate and a strong reductant, and Chiu (2022) showed something similar using polyphosphates, mechanical grinding, and reductants. If the

phosphite is present in the ultramafic/mafic materials as PO33-, then if extracted with D-labeled solvents, they might expect DXPO3X-3 but not if it is present as HXPO3X-3.

I would not expect the authors to perform these experiments as the ICPMS instrumental facilities could not make this determination, but if they provide some further information on how to tell apart different ions present in the rock, and what that might mean for the origin of the phosphite, that would be helpful. Additionally, discussion of the reduction of phosphate to phosphite from a condensed phosphate intermediate may illustrate that reduction is feasible.

The paper's discussion of the origin of pyrophosphate is sufficient. I agree that the formation of pyrophosphate from incompatible phosphate (e.g., as part of glass) is reasonable.

Response: We thank the reviewer for this thoughtful and helpful comment. First, we would like to state that in the revised manuscript, we have explored the role of other parameters such as mantle redox and phosphide presence along with temperature as potential control of P(III) occurrence in the studied samples. We agree that our analytical approach identifies P(III) after aqueous extraction, but it does not by itself resolve the precise in situ chemical form of reduced phosphorus in the rock prior to extraction. In the revised manuscript, we now clarify this point more explicitly. Specifically, our IC–ICP–MS measurements quantify dissolved P(III) in the EDTA–NaOH extract, but they do not distinguish whether the reduced P was originally present in the rock as a protonated phosphite species or a less protonated phosphite ion. In the revised manuscript, we now frame the measured phosphite as evidence for a reduced-P reservoir in the rocks, while avoiding overinterpretation of the exact in situ speciation.

We also agree that the manuscript should say more clearly how the origin of phosphite could be tested in future work. In response, we have expanded the discussion to note that mineral-scale or phase-specific approaches would be required to distinguish among possible origins, including direct in situ spectroscopy, analysis of melt inclusions or glass-rich domains, targeted study of Fe-rich or reduced microdomains, and isotopically labelled extraction experiments such as the D-labelled solvent approach suggested by the reviewer. We do not present these as analyses completed in the present study, but as valuable future tests because our current bulk-speciation data cannot discriminate among those possibilities. The reviewer explicitly suggested D-labelled extraction as a way to probe whether the reduced P is present in different protonation states or precursor forms, and we agree this is an informative future direction, though it is far beyond the scope of our study and outside of our technical capabilities.

Finally, we agree that the mechanochemical reduction of polyphosphates into phosphite[11,12] is indeed interesting, however, we did not use any reducing agent while extraction and the extraction was done in the presence of oxygen in the presence of water. Furthermore, we did not detect any such polyphosphates (except PPi). Therefore, we do not think such reduction is relevant for our samples.

3) The paper omitted reference to phosphite in serpentinites rocks (Pasek et al. 2022), which may be an additional source of phosphite on the early earth (as discussed in paragraph starting at line 55). Perhaps this serpentinite phosphite may be related to the findings in the present paper? Could the intrinsic, reduced phosphorus have been present in the precursor rocks to the serpentinites?

Response: In the revised manuscript, we now cite Pasek et al. (2022)[13] and clarify that phosphite reported from serpentinites may be genetically related to the reduced-P reservoir documented here in mafic–ultramafic igneous rocks. Because serpentinites form by hydration and alteration of ultramafic precursor rocks, it is plausible that at least part of the phosphite observed in serpentinites was inherited from reduced phosphorus already present in the precursor lithologies and subsequently redistributed or released during serpentinization. At the same time, we clarify that our present dataset does not resolve whether serpentinization simply preserves or remobilizes an existing reduced-P

inventory or whether it may also contribute to additional reduced-P formation during alteration. We therefore discuss serpentinites as a complementary reservoir of reactive phosphorus that is consistent with, rather than separate from, the magmatic reduced-P reservoir identified in this study. These revisions are now included in Lines 209-210 and 226-229 of the revised manuscript.

4) The model employs data from Ritson et al. (2020) for the UV oxidation of phosphite. Notably, the short oxidation timescale (<1 day) is in the presence of H2S/HS-. In the absence of sulfide, oxidation timescales are longer. This should be noted even if it doesn't significantly change the findings of the paper.

Additionally, the paper uses the oxidative half-life for Fe(H2PO3)2 for the value of 'dark oxidation'. This was established experimentally using a ferrous phosphite salt that contained water. The oxidation rate of phosphite in water is not well constrained, though Herschy et al. (2018) present a half-life of 103 years in water in air in the absence of UV. In anoxic conditions this may be a lot longer. The authors may want to consider a case where "Pdark_ox" is set to zero, or some other small number, given this large unknown.

Response: We agree that oxidation half-lives of P(III) are uncertain; however, ferruginous anoxic conditions are particularly relevant to the early Earth. Therefore, the half-life determined by Herschy et al. (2018)[14] under these conditions is the best option. Any other value would introduce more uncertainty. We would therefore prefer to keep that value in the model. An oxic endmember is not relevant to our study, because by the time the ocean became oxygenated, life was present and active and further impacting the behaviour of P-species in the ocean[15]. The fluxes for biological processes are unknown. The focus of our study is on the prebiotic ocean, when phosphite-release from oceanic crust may have contributed to the origin and earliest evolution of life. We have emphasized this point in the revised text (line 446-450).

Regarding photochemical oxidation, it is correct that the half-life of phosphite would increase in the absence of sulfide beyond a few hours[16]; however, as long as the half-life is substantially shorter than the residence time of water in the ocean's photic zone (which is ca. 28 years, assuming a surface ocean volume (V) of $3.62 \cdot 10^{16}$ m^3 and a downwelling flux (F) of $1.26 \cdot 10^{15}$ m^3/yr, so that the residence time is V/F = 28 years, after Emerson & Hedges (2008) *Chemical Oceanography and the Marine Carbon Cycle*, Cambridge University Press, pp. 174-178)), the phosphite concentration would still drop to zero. Hence, this parameter would not change our conclusions. But we have added this point to the description of the model (lines 434-440).

Minor comment: I might recommend changing the symbol for Pyrophosphate to PPi, as this is commonly used in biochemistry literature. Since there does not seem to be pyrophosphite (denoted as PPi(III)), this should be fine.

Response: We agree that PP_i is a widely used and familiar abbreviation for pyrophosphate in the biochemical literature. In the revised manuscript, we have therefore replaced PP^{5+} with PP_i throughout for clarity and consistency.

Reviewer #2 (Remarks on code availability):

I looked at the code but did not attempt to run it. My expectation is that the code will solve the problem stated but is not broadly applicable to other systems.

Response: The code is designed specifically to reproduce the box-model calculations used in this manuscript and is not intended as a universally applicable framework.

Reviewer #3 (Remarks to the Author):

This paper presents an analysis of phosphorus compounds within mafic and ultramafic rock samples from 15 locations around the world. Using the analytic results from their IC-ICP-MS experimental measurements and a box model based on a seafloor weathering flux model first presented by Sylverson et al, the authors make an argument for the availability of reactive phosphorus on the early Earth and other planetary bodies of interest to astrobiologists.

The method of extracting P-bearing compounds from rock samples using EDTA-NaOH has been widely used in previous studies, as cited in the paper. The IC-ICP-MS method is a relatively new (ca 2023) method developed and tested by several of the co-authors with supporting citations. IC-ICP-MS seems to be a robust method for detecting low limits of phosphorus-species.

The concentrations of P(III) shown in Table 1 for the various types of rocks are high enough (> 0.6 ppm according to Ref 25) that a more well-established corroborating method (e.g., P-NMR, uv-vis) could have been used to confirm those abundances. This would have strengthened the paper. In lieu of these measurements, a brief explanation of why they are not needed in this case would be helpful.

While the key conclusions of the paper rely on some assumptions, particularly with respect to the box model parameters, those assumptions are clearly stated and supported by citations to literature. Overall, the data appears to be robust, the analysis reproducible, and the interpretation reasonable.

Importantly, this paper establishes that reactive forms of phosphorus, especially reduced and polymerized phosphorus species such as phosphite and pyrophosphate, respectively, may be found in mafic and ultra-mafic rock samples that likely occur in meteorites and in the crusts of other planetary bodies. These more reactive forms of phosphorus promote the formation of phosphorus-bearing organic compounds, which would have been key ingredients for the emergence of life.

Moreover, this article establishes that these mafic and ultra-mafic rocks can release significant levels of reduced and polymerized phosphorus species due to weathering of seafloors, when photochemical oxidation is limited (i.e., up to 0.17 μM). The paper provides examples of key extraterrestrial environments where this water-rock interaction could occur and allow higher concentrations of reactive phosphorus to accumulate, including surface rocks on Mars in the past or on the rocky cores of Enceladus and Europa, where ice-layers could shield ultraviolet radiation.

Thus, this paper makes a strong case that mafic and ultra-mafic rocks in the seafloors and hydrothermal vents within prebiotic oceans could have been important reservoirs for reactive phosphorus contributing to the origin of life on Earth.

I recommend publishing the paper with the one minor suggestion made at the end of the third paragraph of this review.

Response: We thank the reviewer for this positive and thoughtful assessment of our manuscript. We appreciate the reviewer's recognition that the analytical data are robust, the assumptions are clearly stated, the interpretations are reasonable, and the study has broader relevance to reactive phosphorus availability on the early Earth and other planetary bodies. We also thank the reviewer for the helpful suggestion regarding confirmation of the phosphite abundances by an additional method. In response, we want to emphasize that the phosphite concentrations reported in Table 1 are estimated concentrations in the solid rock, rather than direct concentrations in the EDTA–NaOH extract solution. In practice, the phosphite concentration in most of the extract is much lower, as a

result, the analyte concentrations present in solution are too low for practical confirmation by less sensitive alternative methods such as P-NMR or UV–vis spectroscopy in this case. For example. the detection limit for NMR is ~200 ppb, which is the upper limit of most of the samples. We therefore relied on IC–ICP–MS because it provides the sensitivity required for low-level phosphorus speciation in these extracts. Our control data shown in Figure 1 is indicative of the robustness of our IC-ICPMS method. We have revised the manuscript to make this distinction between solid-equivalent concentrations and extract-solution concentrations clearer. These revisions are now reflected in Lines 187–190 of the revised manuscript.

Reviewer #3 (Remarks on code availability):

I believe that anyone with an understanding of Python should be able to use this code effectively. I was able to run the code in Google Colab to replicate Fig 3A in a few minutes.

Response: We also thank the reviewer for examining the code and for noting its reproducibility; our intention in providing the code is to ensure transparency and reproducibility of the specific box-model calculations presented in this study, and we now clarify this scope more explicitly in the revised code availability statement and documentation.

References:

1. Ballhaus, C. Redox states of lithospheric and asthenospheric upper mantle. *Contrib. to Mineral. Petrol.* **114**, 331–348 (1993).
2. Frost, D. J. & McCammon, C. A. The Redox State of Earth's Mantle. *Annu. Rev. Earth Planet. Sci.* **36**, 389–420 (2008).
3. Berry, A. J., Danyushevsky, L. V, St C. O'Neill, H., Newville, M. & Sutton, S. R. Oxidation state of iron in komatiitic melt inclusions indicates hot Archaean mantle. *Nature* **455**, 960–963 (2008).
4. Brunet, F. & Chazot, G. Partitioning of phosphorus between olivine, clinopyroxene and silicate glass in a spinel lherzolite xenolith from Yemen. *Chem. Geol.* **176**, 51–72 (2001).
5. Grant, T. B. & Kohn, S. C. Phosphorus partitioning between olivine and melt: An experimental study in the system Mg2SiO4-Ca2Al2Si2O9-NaAlSi3O8-Mg3(PO4)2. **98**, 1860–1869 (2013).
6. Bindi, L. *et al.* Grammatikopoulosite, NiVP, a New Phosphide from the Chromitite of the Othrys Ophiolite, Greece. *Minerals* vol. 10 131 (2020).
7. Nisbet, E. G. *et al.* Uniquely fresh 2.7 Ga komatiites from the Belingwe greenstone belt, Zimbabwe. *Geology* **15**, 1147–1150 (1987).
8. Baidya, A. S. *et al.* Bioavailable phosphite in the surface ocean during the Great Oxidation Event. *Nat. Commun.* **16**, 4825 (2025).
9. Cui, X. *et al.* Phyllosilicate adsorption limited phosphorus bioavailability in early ferruginous oceans. *Nat. Commun.* **17**, 2422 (2026).
10. Baidya, A. S., Gehringer, M. M., Savaniu, C., Heubeck, C. & Stüeken, E. E. Magmatic and thermally produced reactive phosphorus 3.2 billion years ago and its implications for early life. *Commun. Earth Environ.* **Accepted**, (2025).
11. Löwe, P. *et al.* Ortho-phosphite (PO33−): Mechanochemical Synthesis of a Missing Oxoanion and Precursor to Value-Added Organophosphorus Compounds. *ACS Cent. Sci.* **12**, 40–48 (2026).
12. Chiu, C.-W. Reducing the P-Cycle by Grinding. *ACS Cent. Sci.* **8**, 303–305 (2022).
13. Pasek, M. A. *et al.* Serpentinization as a route to liberating phosphorus on habitable worlds. *Geochim. Cosmochim. Acta* **336**, 332–340 (2022).

14. Herschy, B. *et al.* Archean phosphorus liberation induced by iron redox geochemistry. *Nat. Commun.* **9**, 1346 (2018).

15. Boden, J. S., Zhong, J., Anderson, R. E. & Stüeken, E. E. Timing the evolution of phosphorus-cycling enzymes through geological time using phylogenomics. *Nat. Commun.* **15**, 3703 (2024).

16. Ritson, D. J., Mojzsis, S. J. & Sutherland, J. D. Supply of phosphate to early Earth by photogeochemistry after meteoritic weathering. *Nat. Geosci.* **13**, 344–348 (2020).

We thank the reviewers and the handling editor for their final comments on the revised manuscript. We have now addressed these issues and provide below our point-by-point responses.

Reviewer #1: Remarks to the Author

Comment:
Abu Saeed Baidya et al. have adequately addressed the major concerns in my previous comments. In particular, the statement regarding igneous rocks has been revised in a more appropriate manner, and the explanations concerning the relative proportions of phosphite have been clarified. Overall, this study makes an important contribution to our understanding of phosphorus bioavailability, especially in the context of early Earth environments. I therefore recommend publication after the authors address the following minor issues. Also, I do not need to review the manuscript again and am comfortable leaving the final decision to the editor.

Response:
We thank the reviewer for the positive feedback and for recommending publication after minor revision.

Lines 130–132

Comment:
The sentence appears to be incomplete or unclear. Please revise this statement to improve its clarity and readability.

Response:
We have modified the sentence to improve its clarity and readability. It now reads: "Previous experiments and thermodynamic equilibrium modelling have suggested that, in the presence of Fe(II), elevated temperatures (200–700 °C) favour the reduction of Pi to P(III), thereby increasing the equilibrium P(III)/ΣP ratio[7,21]."

Line 136

Comment:
The rationale for selecting Mo as the representative incompatible trace element is not sufficiently explained. Since several incompatible trace elements could potentially be considered, please clarify why Mo was specifically chosen in this context.

Response:
We thank the reviewer for pointing this out. We have clarified in the revised manuscript that Mo was not used as the sole representative incompatible element. Instead, we compared PPi with more than one incompatible element, including K and Mo. These elements are selected because K is a widely used major-element indicator of incompatible behaviour during igneous processes, whereas Mo provides a trace-element comparison with similarly incompatible behaviour. It is now mentioned in the text.

Lines 220–222

Comment:
The authors have appropriately removed the term "magmatic differentiation," which addresses my main concern. However, the CaO–Al_2O_3 correlation may reflect primary lithological or mineralogical variation, but it should not be used as strong evidence for a magmatic trend or as definitive evidence against alteration. Please revise this statement in a more cautious and descriptive manner, like replacing "indicative of a magmatic trend" with "the positive correlation between CaO and Al_2O_3 is

consistent with preservation of primary igneous/mineralogical compositional variations, rather than being dominated by secondary alteration…"

Response:
Done as suggested. We have revised the statement to describe the $CaO–Al_2O_3$ relationship more cautiously as being consistent with preservation of primary igneous/mineralogical compositional variations, rather than being dominated by secondary alteration.

Reviewer #3: Remarks to the Author

Comment:
The authors have sufficiently addressed the comments of all three reviewers, and I recommend publication of this revised manuscript.

Reviewer #3: Remarks on Code Availability

Comment:
None noted.

Response:
We thank the reviewer for their positive assessment and recommendation for publication.
